# Potential Advantages of Peroxoborates and Their Ester Adducts Over Hydrogen Peroxide as Therapeutic Agents in Oral Healthcare Products: Chemical/Biochemical Reactivity Considerations *In Vitro*, *Ex Vivo* And *In Vivo*

**DOI:** 10.3390/dj8030089

**Published:** 2020-08-07

**Authors:** Martin Grootveld, Edward Lynch, Georgina Page, Wyman Chan, Benita Percival, Eugenia Anagnostaki, Valina Mylona, Sonia Bordin-Aykroyd, Kerry L. Grootveld

**Affiliations:** 1 Leicester School of Pharmacy, De Montfort University, Leicester LE1 9BH, UK; edward.lynch@hotmail.com (E.L.); P16198195@my365.dmu.ac.uk (G.P.); wyman@drwymanchan.com (W.C.); p11279990@my365.dmu.ac.uk (B.P.); eanag1@gmail.com (E.A.); val.mylona@yahoo.com (V.M.); soniareginabordin@gmail.com (S.B.-A.); k.grootveld@btinternet.com (K.L.G.); 2 School of Dental Medicine, University of Nevada, Las Vegas, NV 89106, USA

**Keywords:** oral healthcare products, tooth-whitening products, oral malodor, peroxides, peroxoborate, peroxoborate-polyol ester adducts, reactive oxygen species, oxidants, microbicides

## Abstract

Peroxides present in oral healthcare products generally exert favourable protective activities against the development and progression of tooth decay, plaque, gingivitis, and halitosis, etc. However, despite the high level of research focus on hydrogen and carbamide peroxides as therapeutically active (and tooth-whitening) agents, to date the use of alternative chemical forms of peroxides such as peroxoborates for these purposes has received only scant attention. Intriguingly, peroxoborate and its esters with polyols, such as glycerol, have a very diverse chemistry/biochemistry in aqueous solution, for which there is an increasing amount of evidence that it remains distinctive from that of hydrogen peroxide; such properties include self-associative and hydrolytic equilibria, and their abilities to participate in electrophile- or nucleophile-scavenging, metal ion-complexing, redox and free radical reactions, for example. Therefore, the purpose of this detailed commentary is to evaluate both differences and similarities between the molecular/biomolecular reactivities of peroxoborate species and hydrogen peroxide *in vitro*, *ex-vivo* and *in vivo*. It encompasses brief sectional accounts regarding the molecular heterogeneity of peroxoborates, the release of bioactive agents therefrom, and their oxidative attack on oral cavity biomolecules (the nucleophilic or electrophilic character of these oxidations are discussed). Further areas explored are the abilities of borates and peroxoborates to enhance the solubility of iron ions in aqueous solution, their involvements in free radical biochemistry (particularly the complexation of oxygen radical-promoting transition metal ions by, and antioxidant properties of, peroxoborate-polyol ester adducts), and the specific inhibition of protease enzymes. Further aspects focus on the tooth-whitening, oral malodor neutralizing, and potential mutagenic and genotoxic properties of peroxoborates, along with possible mechanisms for these processes. The abilities of peroxoborates, and peroxides in general, to modulate the activities of inflammatory mediators and vitamins, antioxidant or otherwise, are also explored.

## 1. Introduction

In aqueous solution, oral healthcare and tooth-whitening products containing sodium peroxoborate ([H-O-O-B(OH)_3_]^−^/Na^+^) may liberate hydrogen peroxide (H_2_O_2_), which has well-known and thoroughly established microbicidal and tooth-bleaching actions. However, aqueous solutions of sodium peroxoborate at neutral pH values contain a molecularly-heterogeneous admixture of anionic units (e.g., monomeric, and dimeric species, etc.), in addition to borate and H_2_O_2_ arising from hydrolytic equilibrium processes [1]. Therefore, peroxoborate is structurally diverse, and its promising health effects within the oral environment *in vivo* appear to arise from a composite of those of both H_2_O_2_ and its peroxoborate precursor species. Peroxoborate anion, which has a dimeric structural nature in its crystalline state [2], has seen much use as an eye drop preservative and in many cosmetic product formulations, in addition to various cleaning products and detergents, for example common washing powders [3]. To date, the oral health activities of peroxoborates, along with their ester adducts with polyols, include their applications as effective tooth-whitening agents, and also as an appurtenance to chlorhexidine (CHX) treatment in order to combat adverse extrinsic CHX-induced staining of both tooth surfaces and the tongue [4,5,6,7]. Selected oral healthcare products, e.g., oral rinses and toothpastes, contain therapeutically-active peroxoborate adducts with glycerol and cellulose excipients, and these species may serve to mediate the slow, chemically-controlled release of peroxoborate itself, and subsequently, via equilibration processes in salivary and other aqueous oral environments, H_2_O_2_ too. Lynch et al. [8] explored the ability of an oral healthcare product containing this oxidant, together with its glycerol ester derivatives, to consume salivary biomolecules *in vitro*; these studies revealed that it transformed pyruvate to less acidic acetate, and also was effective at oxidizing the volatile sulfur compound precursors cysteine and methionine to their corresponding disulfide (cystine) and methionine sulfoxide derivatives, respectively. Such observations supported proposals regarding potential mechanisms of action available for peroxoborate anion species and/or their ester adducts, and those for peroxides in general, for example, the caries-limiting removal of stronger salivary carboxylic acids of higher demineralizing potentials, and the neutralization of volatile sulfur compounds (hydrogen sulfide (H_2_S), methyl mercaptan (CH_3_SH) and dimethyl sulfide ((CH_3_)_2_S) responsible for oral malodor.

However, studies focused on the potential microbicidal actions of these agents remain limited. Notwithstanding, to date, Ntrouka et al. (2011) [9] confirmed that one such product (Ardox-X^®^ technology), was effective at killing significantly greater numbers of *S. mutans* present as polymicrobial biofilms adhering to titanium metal discs when compared to results acquired with the other antimicrobial treatments evaluated, i.e., ethylenediamine-tetra-acetate (EDTA), cetylpyridium chloride and CHX; however, H_2_O_2_ and citric acid alone were found to be similarly effective. Moreover, in 2014, this peroxoborate-containing product’s microbicidal activity against oral bacteria, and dental plaque composition in healthy young adults *in vivo* (when applied as an oral rinse formulation throughout a seven-day period), was assessed by Mostajo et al. [10], and these researchers discovered that it expressed a large inter-species variability in its microbicidal activity, with *Prevotella* strains and *Fusobacterium nucleatum* exhibiting the greatest sensitivity; however, *Lactobacillus acidophilus* and streptococci offered a high level of resistance against this treatment. Interestingly, a significant shift in plaque microbiological composition was also noted on application of this treatment, with genus *Streptococcus* and *Veillonella* increasing in count levels, and those of *Corynebacterium, Haemophilus, Leptotrichia, Cardiobacterium* and *Capnocytophaga* decreasing. From this study, it was concluded that this product has the potential for the selective, albeit clinically-significant, inhibition of oral bacteria, with modifications in oral microbiome populations observed following a seven-day rinsing treatment period potentially offering a valuable therapeutic avenue.

Although there is a wide diversity of scientific reports available regarding the favourable oral health and microbicidal actions exerted by H_2_O_2_, or its 1:1 addition product with urea (carbamide peroxide) when present in oral healthcare products (for example, over-the-counter (OTC) mouth rinse products containing 1.5–3.0% (*w/v*) H_2_O_2_, which may facilitate the management and control of plaque, gingivitis, tooth decay and halitosis), and the essential mechanisms associated with these processes [11,12], to date there remains little or no consideration or scrutiny of the use of peroxoborate and its derivatives in such commercially-available formulations. Since the chemistry and biological chemistry of peroxoborates, and their ester derivatives with polyols and carbohydrates, are very highly complex, the purpose of this paper is to critically evaluate possible molecular mechanisms available for their potentially valuable therapeutic activities within the oral environment. These mechanistic considerations are also of much relevance to the microbicidal actions of oral healthcare products containing these agents, and hence their biological chemistry is also evaluated in this context. Such chemical/biochemical activities *in vivo* are also relevant to those featured in the tooth-whitening actions of peroxoborates, and those of peroxides in general.

Primarily, Section 2 of this paper delineates the molecular heterogeneity of peroxoborates in aqueous solution, and how this may affect their chemical reactivities with biomolecules in the oral environment. Section 3 provides a brief outline of the photochemistry of peroxoborate species, which may be of some relevance to their application as teeth-whitening agents, whilst Section 4 explores potential mechanisms involved in the oxidation of biomolecular substrates by these species, with special reference to the active agents featured, including the possible direct delivery of perhydroxyl anion (HO_2_^−^) to biomolecular electrophiles from peroxoborate; generation of significant levels of this active agent from H_2_O_2_ requires high pH values (i.e., > 11), and hence this process is therefore unfeasible in salivary and oral tissue environments without prior pH manipulation. Subsequently, the nature of factors which influence the differential reactivities of peroxoborates and H_2_O_2_ towards functional groups present in a range of organic and biological molecules is reviewed in detail in Section 5, as are the known or proposed mechanisms involved in the oxidations featured. Therefore, this section incorporates sub-sections focused on the reactions of these peroxides, oxidative or otherwise, with thiols and thioethers (of critical importance to the control and management of oral malodor), amines, alcohols and phenols (the former including therapeutically-relevant ester formation for peroxoborates with polyols, etc.), unsaturated compounds, and 5-oxo-carboxylic acids. A final sub-section of Section 5 discusses the interactions and reactions of a peroxoborate-containing oral healthcare product with a wide range of human salivary biomolecules simultaneously via high-resolution ^1^H nuclear magnetic resonance (NMR) analysis, a study which served to reveal the fate of this oxidant (and H_2_O_2_ arising therefrom), in this biofluid, particularly the most important low-molecular-mass, electron-donating biomolecules involved in its consumption. In view of the potential critical importance of hydroxyl radical (^●^OH) generation in therapeutic, cosmetic (tooth-whitening) and toxicological aspects of peroxoborate use, Section 6 encompasses considerations of a series of physicochemical characteristics for the involvement of such species in Fenton and pseudo-Fenton reaction processes, and comparisons of these with those featured in conventional Fenton systems involving H_2_O_2_. These considerations are focused on the maintenance of Fe(II)/Fe(III) ions in aqueous solution-state by the weak complexing activities of borate/peroxoborate species; the accessibility of peroxoborate’s active hydroperoxide (-OOH) function to such ‘catalytic’ metal ions, including their potential complexation by peroxoborate ester adducts at sites remote from this active function; the rates of pseudo-Fenton reactions involving peroxoborate, together with the likely influence of electronic charge, solution viscosity and ionic strength on these processes; and the availability of ^●^OH radical-scavenging antioxidant functions of peroxoborate-polyol and -carbohydrate ester groups. Section 7 then critically explores the use of peroxoborates for tooth-whitening in full detail, in the light of comparative information available for H_2_O_2_ and its 1:1 addition product with urea, carbamide peroxide (CP). Factors considered are the pH-dependence and mechanisms of the tooth-whitening process, along with health and safety aspects of their employment for this purpose at high concentrations, typically, up to 35% (*w/w*) H_2_O_2_ for ‘in-office’, clinically-supervised tooth-bleaching sessions in the USA, but only 1.5–3.0% (*w/w*) for oral rinse agents containing this oxidant as a microbicide. Evidence available for any advantages offered by peroxoborate-containing formulations is discussed. In view of the possible adverse health effects putatively associated with the use of peroxides for tooth-whitening processes at such high levels, Section 8 involves a brief overview of a limited scientific literature report available on the mutagenic and genotoxic potential of sodium peroxoborate when employed in this tooth-whitening context. In view of the abilities of peroxoborates to oxidatively consume both thiol and thioether compounds, in Section 9 we provide an account of a study which evaluated the ability of an oral healthcare product containing this agent to consume malodourous volatile sulfur compounds (VSCs), bacterially-generated agents which are predominantly responsible for oral malodor in humans. Possible mechanisms involved in peroxoborate’s observed ability to significantly depress oral cavity VSC concentrations, and therefore diminish oral malodor, are also discussed. Perhaps surprisingly, evidence available suggests that selected proteinase enzymes may be specifically inhibited by peroxoborate species and not by H_2_O_2_, and therefore the content of Section 10 concerns the mechanisms, and biochemical and therapeutic significance, of these processes. Finally, Section 11 discusses the involvement of inflammatory mediators such as asymmetric dimethylarginine (ADMA), and vitamins such as vitamins C, E and 25-hydroxyvitamin D, in periodontal diseases, and the potential abilities of therapeutically-administered peroxoborate- and H_2_O_2_-containing oral healthcare products to interfere with such activities *in vivo*. Indeed, their roles in causing oxidative/anti-oxidative imbalances in the oral environment are also considered, as are the roles of these vitamins in regulating this redox status *in vivo*.

Rationales for this Commentary:

- To review the applications of peroxoborate species as therapeutic agents for the treatment of oral health conditions such as periodontitis and halitosis, and as tooth-whitening agents;
- To evaluate their clinical successes and possible undesirable side-effects when employed for these purposes, with special reference to their potential modes of action;
- To assess the chemical heterogeneity of peroxoborate species in aqueous solution media and in oral healthcare and tooth-whitening formulations;
- To critically explore and review their chemical reactivities, and the biomolecular mechanisms associated with their favourable clinical effects, when employed as oral healthcare products;
- To compare and contrast their stabilities, chemical reactivities, and positive oral health and tooth-bleaching effects to those obtained with the application of hydrogen peroxide (H_2_O_2_)-alone products;
- Overall: To provide dental clinicians, oral healthcare specialists and scientists with valuable molecular information regarding the clinical and tooth-whitening applications and actions of peroxoborate-containing products as alternatives to well-known and frequently employed H_2_O_2_ formulations.

Null Hypothesis:

- The favourable clinical and tooth-whitening actions of peroxoborates are equivalent to those of equimolar concentrations/doses of H_2_O_2_, and the application-dependent mechanisms of action involved for both agents are identical.

## 2. Molecular Heterogeneity of Peroxoborates in Aqueous Solution: Potential Differential Reactivities with Oral Environment Biomolecules

**Figure 1:**
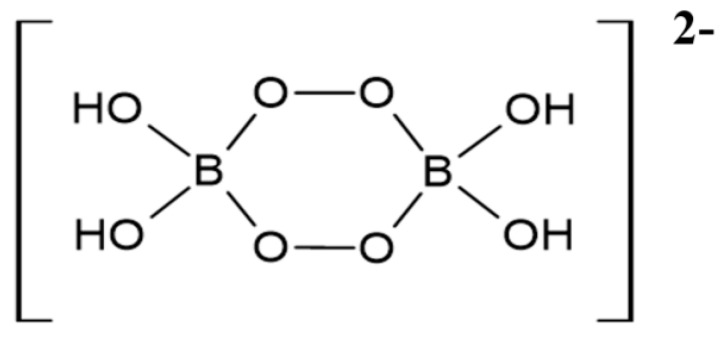
Dimeric structure of the 1,4-diboratetroxane dianion (Structure I).

Firstly, it is of importance to note that the chemistry of peroxoborate in aqueous solution is highly complex, with a realistic total of three or more molecularly-distinct species present at pH values ≤ 9 [13]. Indeed, ^11^B NMR spectroscopy was successfully employed in that study to speciate boron(III) in order to provide evidence for this molecular heterogeneity. Hence, this observation, along with the known formation of its polyhydroxy compound ester derivatives, further complicates peroxoborate chemistry in aqueous solution. Two major solid-state forms of sodium peroxoborate, of empirical formula NaBO_3_.nH_2_O, have n values of 1 and 4, and are classically referred to as mono- and tetrahydrates, respectively. Nevertheless, sodium peroxoborate actually comprises the disodium salt of 1,4-diboratetroxane dianion (Figure 1) [2], and therefore the above mono- and tetrahydrate species represent anhydrous and hexahydrated forms of this structure. Structure I was confirmed by X-ray crystallography [14], along with infra-red (IR) and Raman spectroscopies [15].

In aqueous solution, the precise molecular nature of peroxoborate is critically dependent on the concentration of added peroxoborate hydrate, with monoperoxoborate ([B(OH)_3_(O_2_H)]^−^) dominating amongst all the peroxoborate species present in dilute solutions, and other tri- and tetra-substituted monomers appearing at higher levels. Reaction of borate anions with H_2_O_2_ features rapid equilibria, which are also pertinent to the dissolution and hydrolysis of peroxoborate. In what is described as ‘dilute’ solutions of < 100 mmol./L, Equation (1) depicts the rapid equilibria involved, and this process is supported by ^11^B NMR and Raman spectroscopic investigations [16]. Such ^11^B NMR ‘fingerprint’ experiments have revealed a higher frequency shift of a time-averaged whole system ^11^B resonance, results consistent with these equilibria, which are rapid on the NMR timescale.

[B(OH)_4_]^−^ + H_2_O_2_ ↔ B(OH)_3_ + H_2_O + HO_2_^−^ ↔ [B(OH)_3_(O_2_H)]^−^ + H_2_O (1)

Although in dilute solutions, ‘free’ H_2_O_2_ is found to predominate, some reports have revealed that peroxoborate also contributes towards its reactivity in selected cases [17,18], and this may be ascribable to its ability to deliver the more reactive hydroperoxyl anion (HO_2_^−^) to electron-deficient or other reactants at pH values lower than that required for its production from H_2_O_2_, which has a pKa value of approximately 11.6 (details available in Section 4 below). Hence, selected oral health conditions such as gingivitis may benefit from such behaviour in aqueous environments, and any adverse side-effects arising from the use of these agents are expected to be more tolerable in humans, especially when employed at very high concentrations for tooth-whitening purposes.

Notwithstanding, at higher aqueous solution concentrations the equilibria involved are sufficiently slow for ^11^B NMR resolution and observation at ambient temperatures, and such spectra have demonstrated additional peroxoborate species. Firstly, with an excess of borate, the 1,4- diboratetroxane dianion [B_2_(O_2_)_2_(OH)_4_]^2−^ (I) is produced [16], albeit at relatively low levels in view of its equilibrium with other peroxoborate species; as a sodium salt, this agent has a very low solubility, and hence is readily isolable during peroxoborate manufacturing processes. Secondly, in reaction media containing an excess of H_2_O_2_ over [B(OH)_4_]^−^, the [B(OH)_3_(O_2_H)]^−^ anion along with selected tri- and further tetra-substituted derivatives are generated.

In an aqueous equimolar system containing 200.0 mmol./L borate and 200.0 mol./L H_2_O_2_, the speciation status of H_2_O_2_, [(HO)_3_B(O_2_H)]^−^, [(HO)_3_BOOB(OH)_3_]^2−^, [(HO)_2_B(O-O)_2_B(OH)_2_]^2−^ and [B(OH)_2_(O_2_H)_2_]^2−^ were found to be 92.5, 3.5, 0, 0.5 and 1.55% at pH 7.0, respectively (total peroxoborate-complexed HO_2_^−^ and (O-O)^2−^: 7.6%), and 65, 15, 1, 5.5 and 4%, respectively, at pH 8.0, respectively (total peroxoborate-complexed HO_2_^−^ and (O-O)^2−^: 34%) was reported [19]. Contents of ‘free’ tooth-whitening-active HO_2_^−^ in these admixtures increased nearly 5-fold on increasing the pH from 7.0 and 8.0. However, in Ref. [20], species distribution computations revealed that the solution composition of the active HO_2_^−^ nucleophile in the same aqueous medium was *ca.* 5 and 30% lower than values estimated from the known pKa of H_2_O_2_ (11.6) in the absence of added borate, at pHs of 7.0 and 8.0, respectively, and *ca.* 10% lower at a physiological pH of 7.4. These observations clearly confirm that the generation of the above peroxoborate species (predominantly [(HO)_3_B(O_2_H)]^−^, with smaller quantities of [(HO)_3_BOOB(OH)_3_]^2−^ and [(HO)_2_B(O-O)_2_B(OH)_2_]^2−^) requires HO_2_^−^ anion, and this uptake removes that available in its ‘free’ form in solution. Indeed, if the incorporation of two ‘adducted’ HO_2_^−^ anions into [(HO)_2_B(O-O)_2_B(OH)_2_]^2−^ and [B(OH)_2_(O_2_H)_2_]^2−^, with only one in the remaining two peroxoborate species, is taken into account, the above reductions observed in HO_2_^−^ concentration correspond to the total percentages of peroxoborate species formed. As expected, this reduction in solution HO_2_^−^ level observed was critically pH-dependent. Therefore, this information strongly supports the hypothesis that borates, as peroxoborate species, conserve and deliver the strongly nucleophilic HO_2_^−^ oxidant to suitable scavenging substrates at neutral or near-neutral pH values.

In view of the above considerations, each of the above aqueous peroxoborate species will be expected to have differing reactivities towards biomolecules, and therefore their relative levels present in oral healthcare products may influence their favourable oral health effects. Likewise, the generation of peroxoborate esters with diols, polyols and carbohydrates will also markedly influence peroxoborate reactivity; indeed, such esters may conveniently act as storage reserves for these agents, which, in turn, also store and prolong the actions of more active oxidizing peroxide equivalents.

## 3. Peroxoborate Photochemistry

Rey and Davies (2006) [21] investigated the kinetics of the photochemically-induced decomposition of H_2_O_2_ in borate buffer solutions as a function of reactant concentrations and pH (Equation (2)), and discovered that borate effectively suppressed this photodegradation process at the higher pH values studied. These results were concordant with the generation of mono-peroxoborate and mono-peroxodiborate anions ([B(OH)_3_(O_2_H)]^−^ and [(HO)_3_BOOB(OH)_3_]^2−^ respectively), with thermodynamic equilibrium constants for their formation being 2.0 × 10^−8^ for [B(OH)_3_(O_2_H)]^−^ [13], and conditionally-dependent values of 1.0 or 4.3 for [(HO)_3_BOOB(OH)_3_]^2−^. However, the low quantum yield observed for [(HO)_3_BOOB(OH)_3_]^2−^ was conceivably ascribable to the slower molecular diffusion of the larger ^●^OB(OH)_3_^−^ radical species than that of ^●^OH radical, or alternatively the potential involvement of an intermediate with an oxygen-bridged mono-peroxodiborate cyclic structure. Therefore, it is clear that borate confers an enhanced stability to H_2_O_2_ in aqueous solution through the generation of [HOOB(OH)_3_]^−^ and [(HO)_3_BOOB(OH)_3_]^2−^ adducts. This is consistent with the novel therapeutic properties of peroxoborate-containing oral healthcare products, which may be enhanced and prolonged via the slower, controlled release of active H_2_O_2_, or the lower reactivity of [HOOB(OH)_3_]^−^ and [(HO)_3_BOOB(OH)_3_]^2−^ anions as oxidants towards electron donors, including reductants exhibiting electrophilic or nucleophilic characteristics on reaction.

H_2_O_2_ + *hv* → 2^●^OH (2)

Reference [19] reported that the half-life of peroxoborate in aqueous solution at a temperature of 293 K was as long as 1900 min. but this value was reduced to 280 and only 6 min. at 313 and 340 K, respectively. Moreover, this reference also documented that Fe(II), Co(II) and Mn(II) ions (added as their sulfates) clearly accelerated the degradation of peroxoborate in aqueous solution, as would be expected in view of their ready abilities to participate in Fenton/Fenton-type reactions; a clear logarithmic dependence of the rate of decomposition on added Fe(II) ion concentration was observed within the 1.0–100.0 mmol./L concentration range.

In tooth-whitening episodes (discussed below in Section 7), H_2_O_2_- or CP-containing dental bleaching gels may be activated via the synchronous application of incandescent lamps, light-emitting diodes or, increasingly, selected laser systems, the purpose of this being to decrease the interaction time of the gel on tooth tissues [22]. This is achievable by a process involving promotion of the production of powerfully-bleaching ^●^OH radicals (Equation (2)) through the generation of a photo-thermal incline from the absorption of light by selected pigmented compounds present in such gels. Hence, the above photochemical considerations concerning peroxoborate would be relevant to any corresponding bleaching gels containing this agent in combination with a pigment, and a supporting light or laser source.

## 4. Potential Mechanisms of the Oxidation of Biomolecular Substrates by Peroxoborates: Considerations of Active Species Involved

Peroxoborate itself is a valuable agent for the nucleophilic oxidation of electrophiles (Equation (3)) [1], and such reactions may feature as mechanisms of action putatively available for its use as a valuable therapeutic aid for oral health conditions. For example, its ability to selectively attack key electrophilic biomolecular substrates may be responsible, or partially responsible, for its microbicidal and further oral health actions. Moreover, these reactions indicate that peroxoborates appear to have differential patterns of reactivity than those of free H_2_O_2_, and therefore they may have some selectivity for critical biomolecular targets *in vivo*. Interestingly, both peroxoborate species and their borate/boric acid reaction products serve as effective buffering agents for reaction media, and such considerations may also be applicable to their biological activities *in vivo*.

Since peroxoborate species are less harsh and potentially more specific oxidants than H_2_O_2_ itself, they can also provide benefits in nucleophilic oxidation processes for synthetic organic chemists (Equation (3)). Moreover, at weak alkaline pH values, B(OH)_3_ readily accepts the hydroxide anion (OH^−^) leaving group from the above E-OOH intermediate (Equation (4)).

[B(OH)_3_(O_2_H)]^−^ + E → E-OOH +B(OH)_3_ (3)

E-OOH + B(OH)_3_ → E=O + [B(OH)_4_]^−^ (4)

where E = a biomolecular electrophile

E=O species represent only one example of product classes arising from such nucleophilic oxidations. However, a contrasting example is the epoxidation of olefinic >C=C< units, which introduces three-membered epoxide rings into such substrates (Section 5.4). However, as noted for a range of peroxo-monocarboxylic acids such as peracetic acid, in this case the carbon-carbon double bond acts as a nucleophile, and attacks the more electrophilic oxygen atom in the hydroperoxide function (i.e., that also bonded to the terminal hydrogen, -O-O-H), a process which results in cleavage of its weak -O-O- bond. Strategies for determining the nucleophilic or electrophilic character of substrate oxidations by peroxoborate species are outlined in Section 5.1 below.

However, the pK_a_ value for the hydroperoxide function of a peroxoborate species described as HBO_3_, specifically an (anhydrous) perboric acid (O=B-O-O-H) species, is 7.9 [19], which is much lower than that of H_2_O_2_, (11.6), and is *ca.* 1.3 units lower than the first pKa index for boric acid (9.2). Therefore, it remains possible that such a deprotonated species, specifically >B-O-O^−^, is generated, even at physiological pH values (*ca.* 11 and 24% of the total calculated for pH values of 7.0 and 7.4, for human saliva and blood plasma, respectively). This species may have a similar reactivity to HO_2_^−^, and perhaps also serves as a critical attacking nucleophile for peroxoborate oxidations both *in vitro* and *in vivo*.

Interestingly, although the alkali metal and ammonium cation salts of peroxoborates tend to be formulated as MBO_3_ species, some reports propose MBO_4_ formulae, which may occur in mixed salts of the type MBO_3_.nMBO_4_ [19]; the BO_4_^−^ anion may contain the three-membered ring dioxoborirane species discussed below.

The above considerations will presumably also apply to peroxoborate-glycerol and its cellulosic ester species, provided that they also have available and ionizable reactive hydroperoxyl (-OOH) functions for delivery to biomolecular substrates.

Despite their chemically-similar names, peroxoborate (often also termed perborate) and percarbonate have completely dissimilar molecular structures, with the latter simply being a peroxyhydrate species, and therefore it predominantly behaves in solution as an equimolar admixture of H_2_O_2_ with carbonate anion. However, any small differences noted between the reactivities of H_2_O_2_ and percarbonate solutions are at least potentially attributable to the equilibrium formation of a correctly-named peroxocarbonate species of structure [HO_2_C(O)O]^−^ as shown in Equation (5), although the dominant chemical reactivity of this species is that of H_2_O_2_. Although both peroxoborate and percarbonate oxidants function as valuable sources of H_2_O_2_ in aqueous or predominantly aqueous solutions, the [B(OH)_3_(O_2_H)]^−^ formed on dissolution of peroxoborate is largely activated towards nucleophilic oxidative reactions, whilst in contrast peroxocarbonate is expected to act as an electrophilic oxidant (with nucleophiles (Y:) attacking the peroxo -O-O- bond, Equation (6)), most especially because the reaction driving force is the production of highly stable carbonate anion therefrom [23]. However, there are notable examples of reactions involving the peroxoborate-mediated oxidation of some simple thioether compounds, i.e. R-S-R’, in which the sulfur center acts as a nucleophile which attacks the relatively weak -O-O- bond in peroxoborate, and in such cases this oxidant can be described as having a significant electrophilic character. This was also reported in [23], and these aspects of peroxoborate oxidant characterization with respect to thioether electron donor reactants are discussed in more detail in Section 5.1.

HCO_3_^−^ + H_2_O_2_ ↔ [HO_2_C(O)O]^−^ + H_2_O (5)

[HO_2_C(O)O]^−^ + Y: → CO_3_^2−^ + [Y-OH]^+^ (6)

where Y: = a nucleophile

Delivery of the bleaching-active, mono-deprotonated form of peroxide, perhydroxyl anion (HO_2_^−^) from peroxoborates and/or their ester adducts to HO_2_^−^-scavenging biomolecular electrophiles at lower pH values than those of H_2_O_2_ itself (Equation (3)), is a phenomenon highly worthy of consideration [1], as may be the attack of the >B-O-O^−^ nucleophile noted above. Indeed, for HO_2_^−^, this would permit peroxoborate oxidants to achieve this activity at the neutral or near-neutral pH values of saliva, or perhaps even within acidotic areas of the oral environment (e.g., in carious dentine where the pH value can be as low as 4.5 [24]), although this would depend on the availability of relevant disease-modifying acceptor substrate molecules for this oxidant when acting as a nucleophile. Hence, such peroxoborate species may offer valuable microbicidal advantages over H_2_O_2_ in such environments. As with the potent oxidant hydroxyl radical (^●^OH), HO_2_^−^ is chemically much more reactive as a nucleophile than its parent H_2_O_2_ molecule, and also has much more powerful tooth-whitening actions than its protonated precursor [25,26,27] (its peroxoborate-mediated delivery to likely chromophoric or electrophilic sites in tooth-staining melanoidin ‘browning products’ is discussed in Section 7 below). Importantly, CP- or H_2_O_2_-containing commercially-available teeth-whitening preparations with the highest pH values are more effective bleaching agents [28]. Therefore, in view of its powerful nucleophility, HO_2_^−^ would be anticipated to degrade proteins and other potential biomolecular electrophiles more rapidly and extensively. Such reaction processes include its attack on polymeric melanoidin browning products, which arise from the reactions of dietary reducing sugars with the side-chain amino functions of basic amino acid residues, such as those of lysine and arginine, present in acquired pellicle glycoproteins, and which are considered at least partially responsible for extrinsic tooth discoloration [27]. However, intact H_2_O_2_ is potentially a more potent oxidant (standard reduction potential (ε^o^) values for H_2_O_2_, 2H^+^, 2e^−^/2H_2_O and HO_2_^−^, H_2_O, 2e^−^/3OH^−^ are +1.776 and +0.87 V respectively, Section 6.4), and more so if converted to the powerfully-reactive ^●^OH radical (ε^o^ +2.7 V) via electron transfer from lower oxidation state transition metal ions in Fenton or Fenton-type reactions (Equations (7) and (8)), which is further considered in Section 6 below.

Fe(III) + e- → Fe(II) (7)

Fe(II) + H_2_O_2_ → Fe(III) + ^●^OH + OH^−^ (8)

However, it is important to consider that in view of the base-catalyzed decomposition of H_2_O_2_, the shelf-life of such products is somewhat limited (Section 7). Stable complexes of borate with H_2_O_2_ (albeit as chemically-stable peroxoborates and peroxoborate esters) in dentifrice formulations such as oral rinses and gels have longer or much longer shelf-lives than corresponding ones containing H_2_O_2_ alone, and this phenomenon arises from the ability of borate to successfully limit the availability of the latter reactive oxidant in aqueous solution.

Notwithstanding, in 2011 Durrant et al. [29] provided a new acumen regarding equilibria and species distributions of peroxoborates in aqueous solution media, and their potential involvements in the oxidative modification of a range of substrates. Indeed, results available from their studies have provided strong evidence that a previously undocumented tautomer of peroxoborate anion, a monocyclic three-membered ring species (dioxaborirane), appears to represent the probable catalytic agent involved in borate-modulated electrophilic reactions of H_2_O_2_ towards thioethers at high pH values. Since it is a tautomer of peroxoborate anion, dioxaborirane is in equilibrium with monoperoxoborate through the incorporation of a water molecule. Despite its anticipated low equilibrium concentrations, for its reaction with (CH_3_)_2_S, it has a very low energy barrier (approximately 2.8 kcal.mol^−1^); in contrast, the same oxidation with monoperoxoborate has an activation barrier of 17.5 kcal.mol^−1^, and this value is 10.1 kcal.mol^−1^ for the uncatalyzed reaction with H_2_O_2_. In view of these data, it was deduced that monoperoxoborate was not likely to act as a catalytic source in this reaction system.

Reducing substrates, as explored in [29], included methyl-*p*-tolyl sulfoxide, hydrazine, dimethyl sulfide ((CH_3_)_2_S), halide, hydrosulfide (HS^−^), thiosulfate (S_2_O_3_^2−^) and thiocyanate anions (SCN^−^). These researchers previously observed an unusual pattern of selectivity and reactivity for borate-catalyzed peroxide reactions with organic thioethers, which contrasts with those observed for H_2_O_2_, and inorganic and organic peracids [30], and this unusual behaviour was also experienced with substituted dimethyl aniline nucleophiles. Hence, overall, this further supported the role of dioxaborirane as the probable reactive intermediate involved in these oxidations, and that the patterns of reactivities and products generated was not ascribable to any latent electrophilicities of the substrates involved. Moreover, density functional theory computations applied to the dioxaborirane mechanistic model were found to support experimental data acquired. However, it should be noted that the involvement of a diperoxodiborate dianion (structure I) concentration term in the rate equation and kinetic analysis was only required at high initial concentrations of borate and H_2_O_2_. According to [31], both this diperoxodiborate species and dioxaborirane arise from a common [B(OH)_3_(O_2_H)]^−^ precursor, the former via a route involving [(HO)_3_BOOB(OH)_3_]^2−^.

## 5. Differential Reactivities of Peroxoborate Species and H_2_O_2_ with Biomolecular Substrates

In view of the above considerations, although the majority of oxidations observed for alkaline peroxoborate solutions are attributable to H_2_O_2_ or its more reactive HO_2_^−^ anion, selected reactions, such as that involving the α,β-epoxidation of α,β-unsaturated ketones, have indicated that a boron(III) species is the major oxidant featured; indeed, equimolar reaction composites of tetraborate and H_2_O_2_ were much less effective [17]. Such observations are therefore likely to arise from the >B-OOH function’s electrophilic character-based oxidizing actions.

However, kinetic investigations of peroxoborate oxidations in aqueous solution have largely been conducted at low pH values; these include biologically-relevant reactions with thioethers (as in the amino acid L-methionine) [32], ascorbate [33] and quinols [34]. For ascorbate, a single electron transfer process is featured. However, for reactions with thioethers and quinols, a protonated peroxoborate cation, [(HO)_2_B(OOH_2_)]^+^, was suggested as an intermediate, with an [H^+^] term appearing in the rate-limiting step. Moreover, the quinol oxidation process, which was conducted in an aqueous acetic acid medium, was found to be first-order with regard to [peroxoborate], zero-order with regard to [quinol], and first-order with regard to [H^+^] [34]. Of major relevance to the current paper, this oxidation was faster than that observed with H_2_O_2_, and degradation of the above [(HO)_2_B(OOH_2_)]^+^ intermediate was suggested as the rate-limiting step.

### 5.1. Reactions with Thiols and Thioethers

In addition to their sequential oxidation to sulfenates, sulfinates and sulfonates (Equation (9)), peroxoborate readily oxidizes powerful electron donor thiols to their corresponding disulfides, as we might expect (Equation (10)), and also thioethers to sulfoxides and sulfones, and these reaction systems are fully consistent with those engendered by H_2_O_2_ alone [1] *loc. cit.*, [35,36,37]. Indeed, it serves as an excellent reagent for such processes in synthetic organic chemistry. These properties are also of much significance in oral health biochemistry and dental aesthetics in general, in view of the molecular nature of the major volatile sulfur compounds (VSCs) responsible for oral malodor [38,39]. Moreover, the amino acids L-cysteine and L-methionine are essential precursors for their generation by enzymes available in pathogenic, anaerobic gram-negative bacteria [39]. Hence, peroxoborate and its ester derivatives present in oral healthcare products such as oral rinses or toothpastes will, of course, be expected to exert favourable actions regarding the control and management of halitosis. Section 9 of the current paper provides an outline summary of results acquired from the first reported application of a peroxoborate-/peroxoborate glycerol ester-containing oral rinse formulation for the treatment of oral malodor.

RSH + n(-OOH) → RSOH → RSO_2_H → RSO_3_H (9)

2RSH + [(HO)_2_B(O_2_H)] → R-S-S-R + B(OH)_3_ + H_2_O (10)

RSH + ^●^OH → RS^●^ + H_2_O (11)

2RS^●^ → R-S-S-R (12)

RSR’ + [(HO)_2_B(O_2_H)] → R-(SO)-R’ + B(OH)_3_ (13)

R-(SO)-R’ + [(HO)_2_B(O_2_H)] → R-(SO_2_)-R’ + B(OH)_3_ (14)

Primarily, peroxides such as H_2_O_2_ may directly oxidize thiol compounds to sulfinate and sulfonates, a reaction system which has now been proven to proceed through a sulfenate intermediate [40]; the process depicted in Equation (9) relates to the case where there is an excess of peroxide present, such as there may be in the oral environment immediately following oral rinsing episodes with a mouthwash product containing such oxidants. However, Equation (10) provides an overall balanced equation for an alternative route for the oxidation of endogenous thiols such as L-cysteine, glutathione, or the VSC methyl mercaptan (all depicted as RSH) by peroxoborate to their corresponding disulfides. However, it should be noted that this reaction does not proceed directly, but also involves ^●^OH radical generated from the degradation of H_2_O_2_ through its exposure to light energy sources such as those used in teeth-whitening (Equation (2)), or its catalytic breakdown via the participation of trace ‘catalytic’ transition metal ions in Fenton or pseudo-Fenton reaction processes (Equations (7) and (8)). A simplified mechanism for this process involves the attack of ^●^OH radical on intact thiols and/or their thiolate anions (RS^−^) to generate thiyl radicals (RS^●^) (Equation (11)), which then combine to form corresponding disulfides (RSSR) (Equation (12)), although it should be noted that, if available, molecular O_2_ may directly add to RS^●^ radical generated in Equation (10), and this may complicate the range of products detectable; however, for this oxidation pathway, the disulfide is most commonly the dominant product observed.

Notably, in synthetic routes and pathways, the peroxoborate- or H_2_O_2_-mediated oxidation of both these reactive sulfur centers is generally much more rapid than it is at other molecular functions, e.g., at hydroxy, amino, olefinic or carbonyl groups, and therefore it is not usually considered vital to protect these. However, although the transformation of thioethers to their corresponding sulfoxides usually requires only one added molar equivalent of peroxoborate, small quantities of sulfones are also generated in such reaction mixtures [1].

In the presence of a large excess of peroxoborate and/or H_2_O_2_ over biological thioethers such as the VSC dimethyl-sulfide ((CH_3_)_2_S), as there may be during the initial stages of oral healthcare product use, the sulfone derivative (R-(SO_2_)-R) is expected to be the predominant product, assuming that the rates of Equations (13) and (14) at physiological temperature and pH are not too restrictive. However, at lower or much lower salivary and oral tissue levels of these oxidants, as may indeed be the case some time following oral healthcare product use, it may be anticipated that the sulfoxide, R-(SO)-R, will represent the major product (via Equation (13)).

In a scientifically elegant study, Gomez et al. (2007) [23] explored a very efficient, chemoselective and environmentally-friendly approach for the oxidation of thioethers (described by them as ‘sulfides’) with peroxoborate or peroxocarbonate, and in particular considered the nucleophilic or electrophilic characters of these oxidants under a variety of reaction conditions. This investigation found that the oxidizing activities of these agents differed significantly, with peroxoborate being more effective in aqueous solutions, and having a preference for nucleophilic oxygen transfer processes, whereas for peroxocarbonate an electrophilic character oxidation was preferential, as noted above in Section 4. This information about the nature of oxidant attack may be classified via the electronic character of oxygen-transfer reactions. One extremely useful probe for such electronic character was proposed by Adam et al. [41], who utilized a mechanistic probe containing both oxidizable thioether and sulfoxide groups (thianthrene 5-oxide); a selective oxidation of the sulfoxide function to the sulfone derivative was characteristic of a nucleophilic attack oxidation, whereas a preferential oxidation of the thioether group to the sulfoxide was classified as an electrophilic oxidation. Therefore, an examination of the nature and distribution of products arising therefrom was found to provide a satisfactory measure of the electronic character involved, and hence whether an oxidant attacks this particular substrate in a preferential nucleophilic or electrophilic manner. Such an approach would undoubtedly provide much valuable information concerning the electronic character of biomolecular substrate oxidations by peroxoborate.

### 5.2. Reactions with Amine Functions and Biogenic Amines

Although oxidation of aromatic amines (anilines) to azobenzenes, nitrobenzenes and other products by peroxoborate is quite an efficient process, that for aliphatic amines remains challenging [35]. Nevertheless, a non-physiological biphasic water/ethyl acetate reaction matrix containing this reactant with N,N,N’N’-tetra-acetylethylenediamine gave rise to the favourable oxidation of some primary aromatic amines to C-nitroso derivatives in satisfactory yield [42].

Notwithstanding, since H_2_O_2_ readily oxidizes the biogenic amine trimethylamine (TMA) to its corresponding N-oxide in a facile manner, it is expected that peroxoborate and its derivatives will react in the same or a similar manner, especially on consideration of the large excess of this oxidant provided by freshly-consumed oral healthcare products over that of salivary TMA, which has a mean salivary level of 90–100 µmol./L, and rarely exceeds 300 µmol./L [43]. This reaction, which involves a concerted oxygen atom transfer [44], is also of some relevance to oral health conditions, since this amine is particularly malodorous, whereas its N-oxide reaction product does not have this characteristic at all. TMA is readily detectable in human saliva by high-resolution ^1^H NMR analysis [43], and although arguably less malodorous than VSCs, its salivary concentration is much greater than those of oral cavity VSCs.

### 5.3. Alcohols and Phenols

#### 5.3.1. Oxidation Reactions

Both primary and secondary alcohols react only very slowly with peroxoborate at ambient or physiological temperatures, and often additional reagents are required to promote these processes. Hence, fortunately this does not appear to significantly interfere with the production of peroxoborate-polyol ester species in oral healthcare products and *in vivo*. However, mono-phenols such as the amino acid tyrosine are considered susceptible to oxidation by both peroxoborate and H_2_O_2_, since one study [35] found that more oxidatively-susceptible hydroquinones reacted with peroxoborate to form their corresponding benzoquinones.

Similar to peroxoborate, H_2_O_2_ alone has only a low oxidizing capacity with respect to the conversion of primary and secondary alcohols to their corresponding aldehydes and ketones, respectively [45]. Therefore, in order to enhance this poor oxidation capacity, a range of transition metal ion complexes, such as those of gold, iron, manganese, molybdenum, tungsten, or bismuth has been employed for the oxidation of alcohols, as have selected organic agents such as 1,3-dibromo-5,5-dimethylhydantoin [45].

#### 5.3.2. Boric Acid/Borate and Peroxoboric Acid/Peroxoborate Esters and Complexes

**Figure 2:**
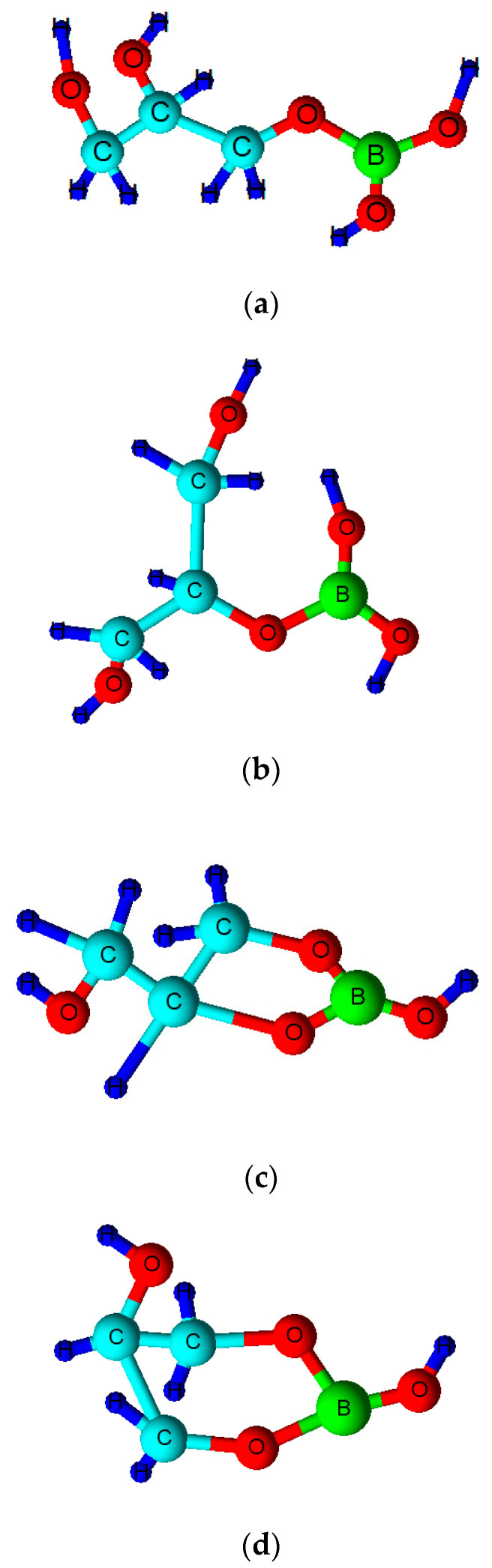
Molecular structures of boric acid/borate monoesters of glycerol. (**a**) *sn*-1(3)-monoester; (**b**) *sn*-2-monoester; (**c**) *sn*-1,2-cyclic diester with 5-membered heterocyclic ring; (**d**) *sn*-1,3-cyclic diester with 6-membered heterocyclic ring. Corresponding peroxoborate esters will contain a peroxo- function (-OOH) in place of one of the two free -OH ones in the case of structures (**a**) and (**b**), and in place of the only free -OH group in structures (**c**) or (**d**).

**Figure 3:**
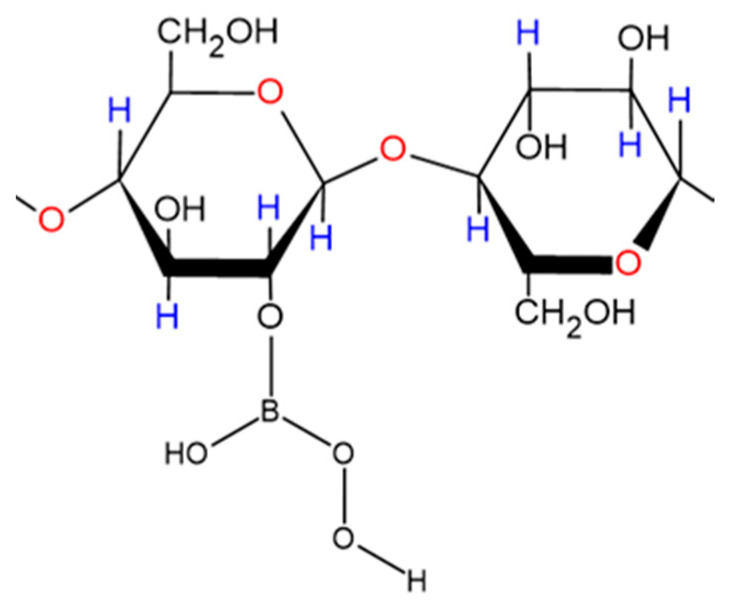
Proposed part-structural representation of a peroxoborate monoester adduct with straight-chain cellulose formed under conditions involving an excess of cellobiose residue concentration over that of peroxoborate.

Borate/boric acid forms ester complexes with polyol species, and an enhancement of the ‘acidity’ of the borate substrate arises from this interaction (i.e., a lowered pKa value for the boric acid/borate moiety). Therefore, such agents are also expected to form in peroxoborate-containing oral healthcare products which also contain glycerol and/or cellulose as excipient ‘filler’ materials, the former of which adds body and consistency. The overall stability of such esters critically depends on the class of diol or polyol involved, and if its -OH functions are oriented in a manner which meets the requirements of tetrahedrally-coordinated B(III), then strong complexes with relatively high stability constants are generated [46]. However, it is also possible for borate to form esters with simple mono-ols such as methanol or ethanol, or to be coordinated by only a single -OH group in a diol or higher alcohol species. The range of possible molecular structures of boric acid/borate esters of glycerol, including both monoester and cyclic diester classes (the latter featuring 5- and 6-membered heterocyclic rings), are shown in Figure 2. A representation of a proposed structural unit of a mono-ester of peroxoborate with cellulose is shown in Figure 3. 

Although electrical conductivity may be employed as a tool to monitor such ester formation, more recently combinations of ^1^H, ^11^B and ^13^C NMR analysis have been utilized to explore the solution equilibria involved. Indeed, ^11^B NMR spectroscopy is adequately suited for this purpose, although it should be noted that B(III) must be present at concentrations sufficient to speciate it, i.e., to permit the electronic integration of its resonances and hence determination of the relative proportions of complex species present in order to compute thermodynamic equilibrium constants for the formation of such adducts.

One study reported in 2006 [47] employed ^11^B NMR spectroscopic analysis to explore the molecular nature and levels of boron-containing compounds in two oral healthcare products containing peroxoborate-glycerol admixtures, one of which was the Ardox-X^®^ technology formulation, which was based on peroxoborate, glycerol and water, with a [glycerol]:[peroxoborate] concentration ratio *ca.* 15-fold. These experiments involved a 600 MHz NMR facility operating at 192.55 MHz for ^11^B and a temperature of 27 °C; samples were analysed with an aqueous pre-calibrated tetraborate solution (0.22 mol./L) present in a capillary insert serving as an external chemical shift reference and quantitative standard. Quantitative determinations of the concentrations of each ^11^B species detectable were performed via normalization of ^11^B degeneracies for each compound. ^11^B NMR spectra acquired revealed that the products investigated contained high levels of both peroxoborate-glycerol ester species and unesterified peroxoborate, the former having a ^11^B resonance *ca.* 1 ppm upfield of the latter. Little or no free boric acid (δ = 19.1 ppm) was found in both products. It was therefore concluded that ^11^B NMR analysis served as a valuable technique for determining the molecular nature of peroxoborate species in aqueous solution, particularly its esters with glycerol and conceivably other polyols, and that the products investigated contained negligible boric acid/borate contents.

Some early investigations of borate esters with diols and carboxylic acids in aqueous solution [46,48,49], and carbohydrates [50], have been performed using ^11^B NMR analysis. Indeed, van Duin et al. [46] studied the pH-dependent stabilities of boric acid/borate esters with glycol, glycolic acid, oxalic acid and glyceric acid using this technique, and found that it provided a valuable analytical probe for structural elucidations and quantitative determinations of a range of such esters in aqueous solution. Furthermore, they stipulated a general ruling that esters of boric acid/borate with dihydroxy compounds have the highest stability at pH values where the overall sum of the ‘free’ esterifying agent’s electronic charge was equivalent to that of the ester formed. A similar rule or consideration may also be applicable to corresponding peroxoborate esters.

### 5.4. Unsaturated Compounds, Including Unsaturated Fatty Acids

Although peroxoborate-induced epoxidations of unsaturated >C=C< function-containing compounds serve as appreciable steps in synthetic organic chemistry routes for the production of epoxides and further products (Equation (15)), these are limited since they are kinetically slow and thermodynamically unfavorable at temperatures below 40 °C. Indeed, higher temperatures and lengthy reaction time periods are often required for the generation of such epoxides and their 1,2-diol hydrolysis products [51]. Notwithstanding, such routes have been successfully employed for the epoxidation of both cyclic and non-cyclic α,β-unsaturated ketones [52,53] and 1,4-quinones [54,55] at very moderately alkaline pH values, i.e., ≥8.5. Moreover, Gupton et al. [56] found that the epoxidations of α,β-enones with peroxoborate was a valuable alternative to the employment of alkaline H_2_O_2_, particularly for reactants with base-sensitive functions such as phenolic groups, and this serves as a good example of the successful achievement of such reactions at a lower pH value than that required for the H_2_O_2_ epoxidation.



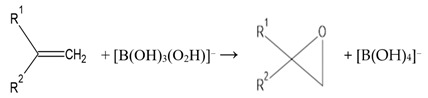
(15)


Furthermore, in human saliva, it is conceivable that peroxoborate may epoxidise unsaturated carboxylic acid anions such as fumarate, a known microbial catabolite. Moreover, it is also possible that epoxidations of mono- and polyunsaturated fatty acids present in this biofluid (as acylglycerols, for example dietary-derived triacylglycerols) will occur via the oxidizing attack of peroxoborate, although the large battery of more highly reactive electron donors available therein, such as pyruvate and L-methionine, will clearly limit these processes.

### 5.5. 5-Oxo-Carboxylic Acids

**Figure 4:**
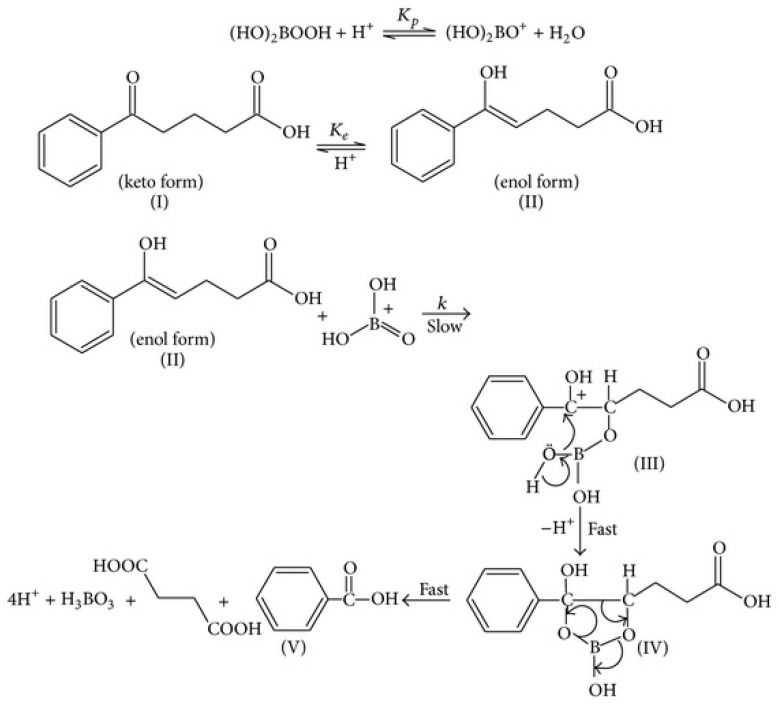
Proposed mechanism for the peroxoborate-mediated oxidation of 5-oxo-acids, which involves a 5-membered ring cyclic intermediate (IV). For this example, benzoic and succinic acids are products. Reproduced from Devi et al. [57] with permission.

Devi et al. (2014) [57] recently investigated the kinetics and mechanism of aromatic-substituted 5-oxo-carboxylic acid oxidization by peroxoborate in an aqueous acetic acid solution; these reductive substrates undergo enolization in acidic media, and the enol form was reported to be the reactive species in these reactions. Benzoic acid derivatives and succinic acid are products of this oxidation. The influence of aromatic ring substitution on these processes was also explored. They found that the reaction was first-order in both 5-oxo-acid and peroxoborate concentrations, and second-order in [H^+^]. Moreover, the reaction rate was accelerated with electron-releasing aromatic substituents and decreased with electron-withdrawing ones. In view of the reaction order with respect to [H^+^], a mechanism involving the [(HO)_2_B=O]^+^ species (Figure 4) as the active peroxoborate oxidant was proposed. Intriguingly, this peroxoborate oxidation was five-fold more rapid than that observed with H_2_O_2_ under the same experimental conditions. Therefore, it is very clear that for 5-oxo-acid reductants, the former offers a more powerful oxidizing power than H_2_O_2_, and from the mechanism proposed by the authors, [(HO)_2_B=O]^+^ forms a 5-membered cyclic boronate ester intermediate with the substrate’s enolic form, which then gives rise to cleavage of the C4-C5 bond, yielding succinic and substituted benzoic acids as products. Of course, such a process would be completely unfeasible with H_2_O_2_ acting as an oxidant.

The ability of metal ions such as Fe(II) and Fe(III) to catalyze this reaction in acidic solution media is discussed below in Section 6.4.

### 5.6. Interaction of a Peroxoborate-Containing Oral Health Product with Intact Human Saliva in Vitro: Evidence for its Mechanisms of Action?

Previous investigations have employed high-field ^1^H NMR spectroscopy in order to perform a multicomponent investigation of the oxidation of salivary biomolecules by peroxoborate present in a tooth-whitening dentifrice formulation, both in the intact human biofluid, and in appropriate chemical model systems containing reactive salivary electron donors [8]. Results acquired from this study demonstrated that added peroxoborate in this formulation gave rise to oxidative decarboxylation of the H_2_O_2_ scavenger and bacterial catabolite pyruvate, a reaction system giving rise to acetate and CO_2_ as products via an oxidative decarboxylation process (Equation (16)). This process was considered to be of favourable benefit towards the control and management of oral health conditions, especially since the pyruvic acid reactant is a much stronger organic acid than the acetic acid product, i.e., it has powerful tooth demineralizing properties, and hence may play a significant role in the pathogenesis of dental caries and other oral health conditions.

CH_3_COCO_2_^−^ + [(HO)_2_B(O_2_H)] → CH_3_CO_2_^−^ + CO_2_ + B(OH)_3_ (16)

Moreover, chemical model system experiments confirmed the oxidative consumption of the salivary electron donors L-cysteine and L-methionine (precursors to volatile sulfur compounds) by oral healthcare product-derived peroxoborate. Indeed, these reductants were oxidized to cystine and methionine sulfoxide, respectively, and this is of much relevance to the ability of such peroxoborate-containing products to prevent or curtail oral malodor via the oxidation of these VSC precursors. Since only the cystine product was detectable in the ^1^H NMR experiments performed on L-cysteine, it appears that the oxidation of this amino acid predominantly involved only the oxidation routes provided by Equations (10)–(12). Of course, VSCs themselves will presumably also be oxidizable by peroxoborate in this manner, although the process for dimethyl sulfide will presumably proceed via Equation (13) and, if peroxoborate is present in excess, subsequently via Equation (14) also. However, for complex multi-biomolecular mixtures such as those found in intact human saliva, there are many alternative peroxoborate scavengers present, including 2-oxo-carboxylate anions such as pyruvate (as noted above) and 2-oxoglutarate, and trimethylamine (Section 5.2), and hence reaction of the peroxoborate oxidant with these scavengers may limit the likelihood and/or extent of Equation (14).

An outline of the ability of a peroxoborate-containing oral healthcare rinse formulation to exert favourable health benefits against halitosis via its effective consumption of gas-phase oral cavity VSCs is available below in Section 9.

## 6. Physicochemical Considerations for the Involvement of Peroxoborate Species in Fenton and Pseudo-Fenton Reactions: Comparisons with H_2_O_2_ as a Reactant

### 6.1. Maintenance of Iron(II)/Iron(III) ion Solubility in Aqueous Solution by Borate and Peroxoborate Species via Complexation Reactions

Boric acid is a very weak acid and has a pK_a_ value as high as 9.14. Therefore, at mean salivary pH values of 7.0, it is predominantly present as boric acid [B(OH)_3_] and is only virtually deprotonated at pH values > 10.5. Borate anion itself serves as a weak complexing/chelating agent for metal ions (i.e. certain lanthanide and actinide ions [58]). Since borate buffer is often employed as a medium to investigate Fenton reaction chemistry, Zhang et al. [59] examined the influence of this buffering system on the ^●^OH radical-mediated degradation of the cyclohexanoate (CHA) scavenger, H_2_O_2_ and a nitriloacetate (NTA) chelator of Fe(II)/Fe(III) ions in a UV light-NTA-Fenton model system. The effects of this buffering agent on the retention of these metal ions in aqueous solution was also explored. These experiments involved comparisons of the effects of a borate buffer of stable pH value (8.00) and MilliQ water media (with a pH value diminishing from 8.00 at time zero to 6.80 on reaction completion). These researchers found that after a 60 min. period, CHA degradation in the borate buffer medium was *ca.* 25% slower than it was in the water medium; H_2_O_2_ decomposition in the borate buffer medium was a little slower than in water within the first 20 min. of equilibrium, but the reverse effect was found during the subsequent 40 min. period; and no overall difference in NTA degradation was noted (97% decomposition was observed in both media). However, most importantly, although a 29% precipitative loss of iron ions was notable in the water-based solvent system, no such loss was observed in the borate buffer solution, and this observation is consistent with its ability to complex/chelate Fe(II) and Fe(III) ions and hence retain them in aqueous solution at pH 8.00. Commonly, Fe(III) products of the Fenton reaction are precipitated as complex hydrated iron(III)-oxide/hydroxide species in the absence of complexing/chelating agents, which can serve to maintain their retention in the reaction solution phase. Moreover, it is known that borate anion can form inner-sphere coordination complexes with iron oxides [60], and hence the generation of a ternary borate-F(III)-NTA complex in these experiments serves as a likely explanation for the results obtained in this study. Of particular note is the possible generation of either free or Fe(II)/Fe(III)-complexed peroxoborate as a further intermediate species.

Evidence provided in the above study also indicated that borate does not scavenge ^●^OH radical, and this indicates the successful liberation of this radical from the metal ion’s coordination sphere. Notwithstanding, the authors of [59] also rationalized that since Fenton reaction-generated ^●^OH radical is bound at the metal ion center as [Fe---^●^OH]^2+^ or [Fe(IV)=O]^2−^species, the co-complexation of borate at this site may hinder the extent and rate of ^●^OH release therefrom. It was also found that the iron ion-complexing potential of borate did not hamper photolysis of the Fe(III)-NTA complex.

Hence, related considerations may also apply to peroxoborate’s role as a potential Fe(II) and Fe(III) ion ligand/chelator, although it should be noted that, since the reactive hydroperoxide moiety remains covalently-bonded to the borate complexant, the availability of released ^●^OH radical for attack on biomolecules from such species is expected to differ significantly from that of Fe(II)-borate complexes’ response to added H_2_O_2_. However, it is conceivable that peroxoborate also has the property of maintaining the solubility of iron ions in aqueous solution reaction media, both in *in vivo* and *in vitro* model systems. Stability constant data for the formation of iron(III)-borate complexes estimated via spectrophotometric titration by Elrod and Kester (1980) [61] were *β_1_ = 1.0 ± 0.2 × 10^−2^ and *β_2_ = 2 ± 1 × 10^−5^ for [FeB(OH)_4_]^2+^ and Fe[B(OH)_4_]^2+^, respectively, at 25 °C and an ionic strength (I) value of 0.68, and therefore it is expected that peroxoborate may also form such mono- and bis-substituted complexes with this metal ion, perhaps with comparable formation constants.

Such inhibitory effects on the generation of ^●^OH radical may serve to account for reports available documenting investigations which demonstrated that: (1) free H_2_O_2_ concentration and ^●^OH radical production from all available -OOH function species present in a peroxoborate-containing tooth-whitening gel formulation (Ardox-X^®^ technology) are significantly lower than those detectable in a 10% (*w/w*) CP product; and (2) that this gel displays a very low level of mutagenicity and does not present risks linked to cytotoxicity [62]. Consistent with the latter observation, only very limited DNA damage was noted in cultured cells treated with a high gel peroxoborate concentration, in contrast to results acquired from corresponding experiments performed with H_2_O_2_ and CP, which revealed marked DNA damaging effects with the ability to give rise to cellular necrosis. The potential mutagenic activities of peroxoborate are succinctly discussed below in Section 8.

### 6.2. Accessibility of Peroxoborate-OOH Function(s) to ‘Catalytic’ Transition Metal Ions? Relevance to ^●^OH Radical Generation

In view of their larger molecular sizes, there may be a more limited molecular accessibility of the -OOH functional group in peroxoborate-glycerol and -cellulosic ester species to Fe(II) ions, which are required for such a pseudo-Fenton reaction system (Equations (4) and (5)), than that encountered by H_2_O_2_.

In addition to HO_2_^−^, the microbicidal (and tooth-whitening) activities of peroxide-containing oral healthcare products potentially arises from the generation of ^●^OH radical, which has a range of potent microbicidal activities, and also exerts powerful bleaching actions towards chromophores responsible for both intrinsic and extrinsic tooth discoloration [27]. One major route for this process involves the interaction of adventitious, ‘catalytic’ iron or copper ions available in the oral environment with H_2_O_2_, or alternative species containing the reactive hydroperoxide (-OOH) functional group (Equations (7) and (8)).

Of course, ^●^OH radical arising from H_2_O_2_ via these Fenton or Fenton-type reactions exerts powerful oxidizing actions towards a wide variety of organic (and putatively also inorganic) biomolecules. As noted above, the generation of ^●^OH radical from H_2_O_2_ is critically dependent on the availability of ‘catalytic’ redox-active transition metal ion complexes (predominantly those of iron(II) or copper(I)) within the oral environment, i.e., those with the capacity to participate in the reaction sequence depicted above). *In vivo*, the molecular status of such iron ion-complexes remains an area of considerable debate, but in hemochromatosis blood plasma [63], and inflammatory knee-joint synovial fluid [64], these appear to present as complexes with the organic acid anion citrate. However, in the absence of a regular supply from dietary sources, salivary concentrations of citrate are limited [41], and it is therefore conceivable that salivary thiocyanate anion (SCN^−^), which is present therein at low millimolar levels [65], serves as a complexant for such ‘free’ catalytic iron ions. The electron in Equation (4) can be supplied by an endogenous reductant (e.g., salivary SCN^−^, thiols, urate, and/or dietary-derived ascorbate). However, ultra-violet (UV) light also induces the production of ^●^OH radical from H_2_O_2_ (i.e., a fragmentation reaction involving cleavage of its relatively weak -O-O- bond, Equation (2)), and this well-known phenomenon serves as the basis for the enhanced tooth-whitening actions attained via a combination of these approaches in tooth-whitening practices. Indeed, H_2_O_2_ (or, more specifically, ^●^OH radical arising therefrom via Equation (5)), and/or its more reactive HO_2_^−^ anion readily decolorize melanoidin browning products (MBPs), which serve as models for those which are at least partially responsible for extrinsic tooth discoloration [18].

Despite this, Miller et al. [66] found that, in view of an ill-defined speciation status and oxidation mechanism, predictions of ^●^OH radical production from Fe(II)/Fe(III)-citrate complex(es) were poor, unlike results they obtained for these metal ions’ well-characterized 1:1 EDTA and diethylenetriamine-penta-acetate (DTPA) complexes; 1:1 iron(III)-citrate complexes are well known to be heterogeneous, including a range of oligomeric/polymeric adducts with co-complexing oxo/hydroxo ligands [67]. Indeed, for these EDTA-/DTPA-chelated Fe(II)/Fe(III) systems, they found that at circumneutral (soil) pH values (6.5-7.5), ^●^OH radical was the only species formed, and kinetic modelling suggested that ‘between-ligand’ differences found in the levels of its production were largely explicable by full considerations of all possible reactions between the redox-active Fe(II)-chelate/Fe(III)-chelate system and reactive oxygen species (ROS). Reactions between the Fe(II)-chelate complex and H_2_O_2_, molecular O_2_ and O_2_^●−^, and that between the Fe(III)-chelate and O_2_^●−^, were found to be of especial importance. However, in the absence of such chelators, ^●^OH radical was undetectable in their analysis system, under conditions in which the predominant oxidant was H_2_O_2_. Hence, the researchers concluded that uncomplexed, aquo-/hydroxo-Fe(II) complexes react with H_2_O_2_ to generate an unknown intermediate which is distinct from ^●^OH. Hence, it appears that ^●^OH radical is only formed when Fe(II) is complexed or chelated by an organic ligand, and to date the oxidant generated from the interaction of Fe(II)-citrate species with H_2_O_2_ remains a source of conjecture.

A quite recent update of evidence available for a wide range of potential mechanistic routes involved in the Fenton reaction system, and the considerable level of debate and controversy surrounding these, is provided by Barbusinski (2009) [68]. Pertinent to the present paper, the mechanism of this process appears to feature the reversible generation of an [Fe(II)---H_2_O_2_]^2+^ and/or [Fe(II)---HO^2−^] intermediate species via the exchange of either intact H_2_O_2_, or HO_2_^−^ anion, respectively, with H_2_O from the metal ion center’s hydration shell. Such an observation may also be relevant to putative pseudo-Fenton reactions involving peroxoborate and/or its deprotonated hydroperoxide function forms, although their esterification with oral healthcare product glycerol may serve to limit its availability for participation in this process. A further, more active intermediate, which is a weak acid with a pKa value of *ca.* 2, and which provides either ^●^OH radical at low pH, and ferryl ion at higher values, has been postulated [68]. The latter ferryl ion route is described as a non-radical pathway.

Hence, the transition state involved in possible hydro-peroxoborate reductions by Fe(II) may be a ferrous-hydro-peroxyborate intermediate involving a single iron-to-oxygen bridging system, just as it is presumed to be for citrate- or adenosine triphosphate (ATP)-chelated Fe(II) complexes [69] (Equation (17), where L represents an endogenous chelated ligand such as citrate or ATP).

As noted in Section 2 above, the peroxoborate species considered here are those predominantly found in aqueous solution on dissolution at neutral pH values, i.e., mainly [(HO)_3_B(O_2_H)]^−^, but also small quantities of [(HO)_2_B(O-O)_2_B(OH)_2_]^2−^ and [B(OH)_2_(O_2_H)_2_]^2−^ [19,20].



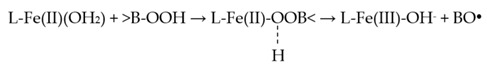
(17)


### 6.3. Potential Complexation of ‘Catalytic’ Transition Metal Ions at Peroxoborate Ester Sites Remote from its Active -OOH Function

The potential complexation/chelation of ‘catalytic’ Fe(II) (and/or Cu(I)) at the -CHOH or -CH_2_OH glyceridic or cellulosic ‘anchor’ sites of peroxoborate-ester adducts is a process which will potentially limit the biological reactivity of any ^●^OH radical arising from reaction of these metal ions with peroxoborate’s -OOH precursory function. This represents an example of intramolecular ^●^OH radical scavenging, or ‘site-specific’ oxidative attack, which has been previously described for other redox-active transition metal ion-complexing molecules, such as the pentose sugar 2-deoxyribose [70]. Indeed, the complexations of Fe(II), Fe(III) and Cu(II) by glycerol [71,72], and cellulosic oxygen donors [73], have been previously investigated in some detail.

In this manner, the generation site of very highly-reactive ^●^OH radical remains remote from critical ^●^OH-scavenging biomolecules such as DNA base moieties and enzymes, etc. Indeed, ^●^OH radical reacts at diffusion-controlled rates (in which the reaction rate is equivalent to the rate of reactant transport through the reaction medium) [74], and therefore does not stray too far from its site of production before being consumed in chemical reactions with any one of many potential bioavailable scavengers. Therefore, in principle, this isolation of ‘catalytic’ iron (and/or copper) ions away from such critical ^●^OH radical-damaging biomolecular sites is potentially achievable by the ability of glycerol- and/or cellulosic-OH groups present in peroxoborate ester species to complex them (possibly with the concomitant release of H^+^ ions therefrom). Such processes may indeed occur within particular oral environments, or at dentifrice treatment time-points where there is a relatively large excess of such esters present over those of oxidatively-susceptible biomolecules. This hypothesis may also serve to explain why oral healthcare products appear not to display any mutagenic and/or genotoxic actions [62], whereas peroxoborate [75] and hydrogen peroxide [76] themselves do.

### 6.4. Considerations of the Potential Rates of Pseudo-Fenton Reactions of Fe(II) Ions with Peroxoborate Species and Their Ester Adducts

The rate of the reaction between Fe(II) with the -OOH group in peroxoborate and its polyol ester species may be significantly slower than that observed with free H_2_O_2_, which has a second-order rate constant (k_2_) value of 5.8 × 10^3^ M^−1^ s^−1^ when the iron(II) ions are present as a complex with citrate (the molecular form in which low-molecular-mass iron ions are postulated to be present as in selected biofluids [60]). Similarly, the rates of reactions of Fe(II) with ‘free’ (non-polyol-esterified) peroxoborates are likely to differ from that observed with H_2_O_2_. To date, an extensive literature review has revealed that there are no published rate constants for the reactions of Fe(II), chelated or unchelated, with aqueous peroxoborate species alone.

However, it is generally accepted that the rate of the reaction of ‘free’, presumably uncomplexed/unchelated Fe(II) (presumably as Fe(II)_(aq.)_) with H_2_O_2_ in biological systems is approximately 5 × 10^2^ M^−1^ s^−1^ [77], which is apparently too slow for significant levels of oxidation to occur *in vivo*. However, as with citrate above, with complexation by physiologically-available complexants and chelators, including ATP, this rate increases by at least one order of magnitude [77].

Moreover, Rush and Koppenal (1991) [78] reported that second-order rate constants for reductions of the large bulky aromatic-substituted cumyl hydroperoxide by Fe(II)_(aq.)_ at low pH, and with the Fe(II)-EDTA chelate at neutral pH (16, and 1.1 × 10^3^ M^−1^ s^−1^ respectively), are lower than those determined for the corresponding Fenton reaction system with H_2_O_2_ (42, and 7.0 × 10^3^ M^−1^ s^−1^ respectively) by factors of 3-6. Larger differences were found on comparison of the rates of cumyl hydroperoxide and H_2_O_2_ reductions by an Fe(II)-pyrophosphate complex at neutral pH value. These researchers also discovered that the Fe(II)-citrate and -ATP complexes react more rapidly with H_2_O_2_ than they do with cumyl- and *t*-butyl hydroperoxide, by factors of 2–5. Interestingly, they surmised that this observation is at least partially explicable by a statistical index of 2, since H_2_O_2_ has two reactive ^●^OH sites, whereas the above alkyl hydroperoxides only have a single site. Indeed, further kinetic evidence strongly indicated that the transition-state complex formed preceding electron-transfer involved only one of the hydroperoxo function oxygen atoms (Equation (17)).

In view of these observations, unesterified peroxoborate would be expected to react more slowly with (unligated) Fe(II)_(aq.)_ than would H_2_O_2_ in view of this transition state postulate. If the peroxoborate is pre-esterified to relatively bulky polyol species such as glycerol or cellulose, most especially the latter, then reductions in rate commensurate with ester ‘carrier’ bulkiness might be anticipated.

In addition to the rates of the above reaction systems, a further important consideration is the relative thermodynamic favorabilities of the reactions of Fe(II) with H_2_O_2_, peroxoborates and their ester adducts. Perhaps the thermodynamic equilibrium constants for these processes are less favourable for peroxoborates and its glycerol and cellulose esters, i.e., is there a lower favorability or extent of transformation of the -OOH group in these agents to ^●^OH radical than there is for H_2_O_2_? In view of perceived complications with its determination arising from peroxoborate’s ready dissociation to borate and H_2_O_2_ in dilute aqueous solution (Equation (1)), there is no information available on its reduction potential. However, as early as 1955, Kern [79] discovered that in a polarographic study of the H_2_O_2_/O_2_ couple, the diffusion current of H_2_O_2_ was much diminished by borate ion, an observation confirming that the oxidizing power of peroxoborate species predominating in aqueous solution significantly differs from that of H_2_O_2_ [80], despite the low values available for their formation constants in aqueous solution [61]. Clearly, the esterification of peroxoborates will also be expected to modify the ε^o^ value of each species adducted in this fashion, but perhaps only marginally so.

Interestingly, the role of added iron(II) ions in catalyzing the peroxoborate-mediated oxidation of 5-oxo-acids (with aromatic substituents present at the 6-position) was recently investigated in acidic aqueous solution [81], and this reaction was found to be first-order in both peroxoborate and catalyst concentrations, and less than first-order in that of the reducing substrate, i.e., the rate saturated at high 5-oxo-acid concentrations when present in excess under pseudo-first-order conditions, i.e., with [5-oxo-acid]_0_ >> [peroxoborate]_0_ >> [Fe(II)]. The rate was also independent of pH value. In view of these results, it was concluded that H_2_O_2_ was the active oxidant species, and that a ternary iron(III) complex with coordinated peroxo- and 5-oxo-acid ligands was generated in the reaction sequence. However, this reaction’s rate was very similar to that observed with equivalent levels of an Fe(III) catalyst in place of Fe(II).

This reaction was triggered by the addition of H_2_O_2_ to an admixture of the Fe(II) catalyst with 5-oxo-acids. presumably in the presence of atmospheric O_2_, since there is no documented record of its exclusion during the reactions performed. Hence it is conceivable that Fe(II) is autoxidized to Fe(III) (Equation 18, a process also generating superoxide anion [O_2_^●−^]), a process which may indeed be stimulated by complexation of the former by the 5-oxo-acid substrate, which in turn may significantly reduce the ε^o^ of the Fe(III)/Fe(II) couple, as noted for iron(II)/(III)-citrate chelates [63,64]. Moreover, if the above O_2_-mediated autoxidation does not take place, or is incomplete, then it is virtually inconceivable that a Fenton or pseudo-Fenton reaction yielding ^●^OH will not occur on addition of H_2_O_2_ to the reaction mixture as specified above, especially in view of the thermodynamic favorability of the equilibrium shown in Equation (1) towards the H_2_O_2_ direction in acidic aqueous solution; this Fenton reaction process may involve 5-oxo-acid-complexed or uncomplexed Fe(II) as a catalyst. Although these researchers did explore the possible generation of ^●^OH radical during the reaction process, a Fenton-type reaction was ruled out since they did not detect any radical species with an electron spin resonance (ESR) study of the reaction solution, and also found that the iron(II)-catalyzed oxidation of 5-oxo-acids was not responsive to the addition of vinyl monomer, and nor did it induce acrylonitrile polymerization, Notwithstanding, it is very important to note that if these tests were conducted following the addition of H_2_O_2_ to the Fe(II)/Fe(III)-5-oxo-acid reaction admixture, then it is not likely that any would be detectable in any case, in view of the probable rapidity of the above Fe(II) autoxidation and/or Fenton/pseudo-Fenton reactions, which are expected to be facilitated by the reductant substrate, and also the extremely rapid scavenging of any ^●^OH radical formed from the latter process by these aromatic function-substituted compounds (via aromatic hydroxylation, or attack on their aliphatic chains [82]).

Fe(II)-L + O_2_ → Fe(III)-L + O_2_^●^^−^ (18)

### 6.5. Availability of Hydroxyl Radical (^●^OH) Scavenging (Antioxidant) Functions in the Glycerol or Cellulosic Moieties of Peroxoborate-Ester Adducts

The availability of further ^●^OH radical-scavenging sites in peroxoborate-polyol esters, for example any available ^●^OH radical-scavenging glyceridic- or cellulosic-OH functional groups in such adducts (Equation (19)), unlike H_2_O_2_ itself, is another important factor for consideration. Indeed, the aggressively-reactive and highly toxic ^●^OH radical may arise from biological Fenton reactions *in vivo* (Equation (8)), and may cause damage to critical biomolecules, albeit if its tissular distribution and actions are limited to the oral environment. For example, it can oxidatively modify DNA structure, along with those of critical enzymes. Hence, such peroxoborate-ester polyol moieties may serve to offer protection against such potentially deleterious attacks. In this context, they are acting as ‘antioxidants’. It has been long established that glycerol is a very powerful ^●^OH radical scavenger, and this reaction generates formaldehyde as a product. In order to explore this, in 1994 Rashba-Step et al. [83] generated glyceryl radical species from the photolysis of H_2_O_2_, the xanthine oxidase-mediated oxidation of xanthine in the presence of an iron ion catalyst, and a nicotinamide adenine dinucleotide phosphate (reduced form, NADPH)-dependent microsomal electron transfer system also containing the Fe(III)-EDTA chelate, and confirmed that ^●^OH radical was indeed involved in this process. Moreover, ESR experiments involving the 5,5-dimethyl-pyrroline N-oxide (DMPO) spin trap also verified that glyceryl radical species were produced from the reaction of ^●^OH radical with the glycerol substrate. Therefore, selected oral rinse and tooth-whitening gel products containing admixtures of glycerol with peroxoborate (of molar ratios typically > 10:1), and carbohydrates such as cellulose, may offer an enhanced level of protection against any adverse health effects exertable by peroxide species in view of the ability of such polyols to effectively scavenge DNA-damaging ^●^OH radical. Notwithstanding, the tooth-whitening actions of peroxoborate and H_2_O_2_ are potentially ascribable to the ability of this radical to attack and decolorize brown-colored melanoidin and/or iron- and tin-sulfide staining sources, but this process should, of course, be strictly targeted on and limited to the tooth surface only, so that adjacent oral soft tissues remain unexposed to these oxidants, irrespective of whether it is clinically-applied ‘in-office’, or self-applied by patients at home.

-CH_2_-OH + ^•^OH → -CH_2_O^●^ + H_2_O (19)

### 6.6. Potential Influences of the Electronic Charge and Medium Viscosity of Peroxoborate/Peroxoborate-Ester Derivatives, and Salivary Ionic Strength, on Their Biochemical Reactivities

The overall electronic charges of peroxoborates and their glycerol and cellulosic ester adducts may also determine their chemical reactivities and oxidizing capacities in the oral environment; however, although boric acid is only a weak acid, it generates the anionic [B(OH)_4_]^−^ anion in aqueous solution. Similarly, [B(OH)_3_(O_2_H)]^−^ arising from the dissolution of sodium peroxoxborates in aqueous solution (Equation (1)) is also negatively-charged, as may indeed be its ester species with polyols such as glycerol and cellulose. This, in turn, may affect its reactivity with Fe(II) complexes putatively available in the oral environment for Fenton-type reactions, perhaps as negatively-charged citrato-, or various thiocyanato-Fe(II) complexes, which will also have negative electronic charges with higher degrees of SCN^−^ ligation. However, if ‘free’ peroxoborate, or the peroxoborate moiety of its polyol esters, complex salivary Ca^2+^ or Mg^2+^ ions, then depending on the number of ligated oxidants at the metal ion co-ordination sphere, this charge may be neutralized or become positive, irrespective of whether polyol esterification is involved or not (calcium borate has a water solubility of *ca.* 1% (*w/v*)). Similar electronic charge switches may occur if peroxoborate and its esters weakly bind traces of ‘catalytic’ Fe(III) ions, as indeed borate does [61].

Nonetheless, if such peroxoborate oxidants remain anionic, and if Fe(II) is also present as a negatively-charged complex or chelate at physiological pH values in human saliva or at tooth discoloration sites, an electrostatic repulsion between these two Fenton system reactants would be expected, as opposed to the Fe(II)/H_2_O_2_ model, where H_2_O_2_ is uncharged at physiological pH values. Such an electronic repulsion might be expected to give rise to a lowered rate of reaction between these similarly-charged species.

One pertinent observation involves the oxidation of ascorbate by peroxodisulfate in a micellar medium [33]. Indeed, at H^+^ concentrations > 9.6 × 10^−4^ mol./L (provided by H_2_SO_4_ or HClO_4_), the rate of this oxidation was found to decrease, and this effect was explicable by the repulsive interaction of negatively-charged lauryl sulfate micellar species with the ascorbate coanion HA^−^.

MBPs, which are at least partially responsible for extrinsic tooth discoloration [27], have a high level of molecular diversity, and contain peroxide oxidant-susceptible carbon-carbon double bonds (>C=C<) in their molecular structures. However, a series of investigations have reported that these complex and highly variable structures are negatively-charged, in both chemical model systems and in food products [84]. Indeed, Kwak et al. [85] demonstrated that melanoidins arising from the reflux of glucose with lysine were separable into 14 component bands throughout the 3.50–4.85 pH range, an observation providing evidence that these species are negatively-charged at neutral pH. However, it should be noted that the class of amino acid employed for MBP development determines their anionic characteristics. Hence, these anionic charge properties may influence the direct delivery of tooth-whitening HO_2_^−^ species to these target sites by [B(OH)_3_(O_2_H)]^−^ or its polyol ester adducts.

However, whatever the charges of the reacting species involved, when they are of the same sign, increases in ionic strength will enhance the rate, and vice-versa in cases in which they are of opposite sign [86]. Considerations of medium ionic strength (I) indices have been found to be important in biochemical processes involved in the oral environment. For example, Yao et al. [87] found that the characteristics of papain hydrolysis in stimulated whole human salivary films was critically affected by I values. Moreover, since citrate, phosphate and tartrate are often present at quite high levels in oral healthcare formulations such as mouth rinses, the infiltration of these anions into the oral environment from such sources immediately subsequent to product use will markedly, albeit only transiently, increase the I value of human saliva. Moreover, it should also be noted that such anions, e.g., citrate, interact with peroxoborates to form ester adducts, and it has been shown that peroxoborate hinders the rate of calcium ion abstraction from hydroxyapatite by citrate and tartrate solutions [19], *loc. cit*. The effects of these anions may arise from their esterification to peroxoborate species, as observed with polyols and hydroxycarboxylic acid anions (Section 5.3.2 and references cited therein), or they may simply cause a disturbance to peroxoborate-borate/H_2_O_2_ equilibrium processes.

A further consideration is the relatively high viscosity of glycerol-containing (glyceridic) and, consequently, peroxoborate-glycerol ester adducts in selected oral healthcare products, for example tooth-whitening gels and other application products. Although such viscogens will only be present within the oral environment or, where appropriate, on tooth surfaces during the early stages of treatment protocols when applied as visco-elastic gel formulations, they may limit the accessibility and hence reactivity of Fe(II) towards this oxidant’s -OOH function, which, in turn, may be expected to inhibit the generation of tooth-whitening ^●^OH radicals. Moreover, the large excess of glycerol or other polyols present at these treatment time-points will also serve to scavenge any ^●^OH radical generated (as noted above in Equation (8)), and in a thermodynamic context, will therefore successfully compete for this radical species, a process which may also suppress its attack on critical endogenous biomolecules. It is well known that solvent viscosity is also an important consideration for the determination of reaction rates [86]. Indeed, in highly viscous environments or solvent systems, reactant solutes diffuse more slowly than they do in less viscous ones, i.e., they collide less rapidly per time unit, and hence the rates of chemical reactions markedly diminish with increasing matrix viscosities.

## 7. Peroxoborate Species as Alternative Tooth-Whitening Agents to H_2_O_2_

### 7.1. H_2_O_2_ and Carbamide Peroxide (CP) as Agents for the Removal of Unaesthetic Tooth Stains

H_2_O_2_ is frequently employed as a dental tooth-whitening agent [88,89], and bleaching products available contain it at low concentration for ‘at-home’ sessions, or alternatively at higher levels for ‘in-office’ applications [90,91,92]. It is, however, readily degraded by a range of chemical reaction sequences, which may be induced or affected by its exposure to incident light, reaction/storage temperatures, pH values and/or electron transfer from catalytic, redox-active transition metal ion complexes, etc. [89,93].

CP, a hydrogen-bonded 1:1 addition complex of this oxidant with urea, is typically also applied for tooth-whitening purposes at levels ranging from 3-20% (*w/w*), and a 10% (*w/w*) content of this complex liberates 3.6% (*w/w*) H_2_O_2_ [90]. Predominantly, such CP-containing tooth-whitening products also contain carbopol polymer or glycerol; the former effectively gives rise to the slow release of H_2_O_2_, but this does not alter treatment effectiveness. CP preparations have marginally acidic pH values.

### 7.2. Dependence of the Bleaching Actions of Peroxides on pH

The perhydroxyl anion (HO_2_^−^) is often erroneously described as a ‘reactive free radical’ in the oral health field literature, but nevertheless can still be described as a ROS. Facilitation of the production of this active species may be achieved via the alkalization of H_2_O_2_ bleaching formulations (Equation (20)), but in view of its time-dependent decomposition at pH values > 8.0 [94], it is very important that such a measure is taken immediately before use, as in the CP-containing product evaluated in [28], which has its pH value adjusted by an amino-alcohol product activator. Optimal pH values for this process are 9.5-10.0 [95,96]. Although the pKa value for the mono-deprotonation of H_2_O_2_ is *ca.* 11.6 [1], modification of product pH values as high as or higher than this value severely diminishes the quantity of H_2_O_2_ available for bleaching activities [97], and drastically reduces product shelf-lives. The most frequently employed concentration of H_2_O_2_ employed in the USA is typically 35% (*w/w*) [7].

### 7.3. Consideration of Potential Mechanisms Available for Peroxide-Mediated Tooth-Whitening Processes

The enhancement of positive tooth-whitening results obtained with increasing pH values, which is explicable by the deprotonation of H_2_O_2_ to the perhydroxyl anion (Equation (20)) [98,99,100,101]), may then give rise to the generation of ^●^OH and HO_2_^●^ radicals (Equation (21)), which serve as the active bleaching agents involved, or at least markedly contribute towards the tooth-whitening activities of peroxide-based tooth-whitening agents. Further HO_2_^●^ may then be generated via the interaction of ^●^OH with excess H_2_O_2_ (Equation (22)). Hence, HO_2_^−^ appears to act as the primary key species responsible for teeth-bleaching outcomes arising from the application of products containing such agents onto the tooth surface. As expected, the tooth-whitening effect observed is incrementally enhanced with increasing pH values of the applied products [97].

H_2_O_2_ + OH^−^ → HO_2_^−^ + H_2_O (20)

HO_2_^−^ + H_2_O_2_ → HO_2_^●^ + ^●^OH + OH^−^ (21)

^●^OH + H_2_O_2_ → HO_2_^●^ + H_2_O (22)

As noted above, extrinsic tooth discoloration in older adults is considered to be partially attributable to MBPs formed from Maillard reactions. This highly complex reaction system primarily features condensation reactions of carbonyl compounds and reducing sugars with available amino functions of free amino acids, peptides and proteins, and glycoproteins of the acquired pellicle serve as likely substrates for this undesirable chemical process [27]. Hayase et al. (1984) [102] investigated the decolorization of non-dialysable melanoidins generated from a glycine-glucose model system by treatment with H_2_O_2_ directly, or indirectly via its generation from a glucose oxidase enzyme-substrate reaction. With the latter enzymatic system, brown-colored non-dialyzable melanoidins were decolorized to 65%. However, their treatment with H_2_O_2_ (final added concentration 6.72% (*w/v*)) successfully decolorized these melanoidins to levels of 64 and 97% at pH values of 7.0 and 10.0, respectively, and this observation serves to confirm that the HO_2_^−^ anion is a more effective tooth-whitening agent than H_2_O_2_ itself. Moreover, as expected, the mean molecular mass of such melanoidins was reduced from 5300 to 3500 following H_2_O_2_ treatment. Major reaction products detectable in an ether-soluble fraction obtained from melanoidins treated with H_2_O_2_ at alkaline pH were 2-methyl-2,4-pentanediol, N,N-dimethylacetamide, phenol, acetic acid, oxalic acid, methylpropanedioic acid, propane-dioic acid, 2-furancarboxylic acid, butane-dioic acid, 2-hydroxypropanoic acid, 2,5-furandicarboxylic acid and 5-(hydroxymethyl)-2-furancarboxylic acid. However, the major product of this oxidative degradation which was identified in the aqueous phase was glycine, in *ca.* 2% yield.

Interestingly, dark-colored iron(II)- and iron(III)-sulfides, and/or sulfido complexes, are also partially responsible for extrinsic tooth discoloration [27], and hence it is not inconceivable that peroxides may interact with the former in order to generate ^●^OH radical site-specifically *in vivo*, a process facilitating their tooth-whitening actions via a molecularly ‘self-destructive’ mechanism.

### 7.4. Health and Safety Considerations for H_2_O_2_ and CP Tooth-Whitening Formulations: Potential Adverse Health Effects

Despite their effectiveness, the inappropriate or unqualified use of H_2_O_2_ or CP for tooth-whitening episodes has revealed a series of safety issues and adverse health effects [103,104], and these include: (1) tooth sensitivity, this being a common side-effect; (2) cervical root resorption, which may arise from internal bleaching, and is often noted in teeth treated with the application of a thermo-catalytic whitening strategy; (3) the erroneous employment of these oxidants < 24 h. prior to the application of resin-based materials as restorative treatments; and (4) the potential genotoxic and tumor-promoting actions of such H_2_O_2_-containing tooth-whitening products. It is therefore advised that such products of high H_2_O_2_ content should not be applied without gingivae protection, which affords circumvention of gingival tissue or mucosae exposure. Similarly, H_2_O_2_-containing products should not be used in patients with damaged or diseased tissues.

### 7.5. Tooth-Whitening Activities of Peroxoborate Species and Their Polyol Esters

For the purpose of its tooth-whitening applications, sodium peroxoborate is available in three apparently distinct forms, not only the monohydrate and tetrahydrate derivatives, but also a trihydrate [105], which differ in their weight-per-weight whitening efficacies [106] in view of the higher weight proportion of active hydroperoxide functions present in the monohydrate, and sequentially smaller levels in the tri- and tetrahydrates. According to [107], a 2 g/1.0 mL mixture of sodium peroxoborate exerts a bleaching action equivalent to that of a 16.3% (*w/w*) H_2_O_2_ preparation, and from these data it therefore appears that the latter oxidant is much more effective than the former.

However, since peroxoborate is potentially less hazardous and more stable than H_2_O_2_, it appears to offer some major benefits when used as a tooth-whitening technology. Nevertheless, to date, there is only a limited number of *in vitro* investigations which have found that sodium peroxoborate solutions in water, and in combination with 3 and 30% (*w/w*) H_2_O_2_, or 10% (*w/w*) CP, are all active when applied to the internal bleaching of non-vital teeth [108,109,110]. Hence, although CP and H_2_O_2_ are more powerful tooth-whitening agents than peroxoborate, the latter displays improved penetrating properties over those of CP; although all bleaching agents were able to penetrate from the pulp chamber to the external root surface, a combination of peroxoborate with CP was found to be the most effective in this context [111]. This study also found a direct correlation between penetration potential and the presence of tooth-whitening oxidants, and also concluded that aqueous solutions of peroxoborate acted as the least aggressive bleaching agent applied. Intriguingly, application of admixtures of peroxoborate with CP appear to exert synergistic actions towards the bleaching of blood-stained teeth, i.e., over and above the additive effects of both agents applied individually and consecutively [112]. This study is further discussed below.

In 1993, Weger et al. [113] explored the influence of a range of sodium peroxoborate products on the pH values of bleaching agents, and for this purpose they individually mixed the mono-, tri- and tetrahydrates of sodium peroxoborate with 0, 10, 15 or 30% (*w/v*) H_2_O_2_ at a powder: liquid ratio of 2.0 g:1.0 mL. These researchers found that the pH of these admixtures significantly increased with increasing added H_2_O_2_ concentrations, and such pH values were also dependent on the peroxoborate water of crystallization content and time of post-mixing measurement. Therefore, they recommended that the pH values of such tooth-bleaching formulation mixtures should be strictly monitored prior to use in order to circumvent possible post-bleaching root resorption problems. However, little or no explanation of the association between this water of crystallization content and medium pH value was offered.

Interestingly, in 1994 Lewinstein et al. [114] discovered that although 30% (*w/w*) H_2_O_2_ significantly reduced the microhardness of both enamel and dentin, treatment of these matrices with admixtures of peroxoborate and H_2_O_2_ did not exert such effects at experimentally-related temperatures (37 and 50 °C) and time intervals, so these composites appear to successfully circumvent such side-effects and hence offer health and safety advantages over high H_2_O_2_ content formulations. It was also concluded that bleaching practices with such high H_2_O_2_ concentrations should be limited, or only used with extreme caution.

The abilities of CP (16% (*w/w*)) and sodium peroxoborate, together with an admixture of these two agents, to whiten non-vital, blood-discolored teeth *in vitro* were investigated in detail by Valera et al. (2009) [112], and they concluded that all three treatments exhibited similar bleaching capacities at time-points of 7, 14 and 21 days when tested against a negative distilled water control group (*p* < 0.05). However, as noted above, the composite CP and peroxoborate treatment option was found to facilitate the return to their original shade indices in all teeth evaluated after a 21-day duration (bleaching agents were replaced twice at seven-day intervals for the total 21-day period). Therefore, as noted above, these results indicated that peroxoborate, in combination with CP, exerts a greater tooth-bleaching activity than that found for CP alone; in addition to the above tooth-penetrating properties of peroxoborate, such an advantage may arise from its ability to stabilize and hence enhance the longevity of H_2_O_2_ action, and it is possible that this is achievable through the continuous equilibrium exchange of the latter oxidant with peroxoborate (Equation (23)).

(HO)_2_B-O-O-H + H-*O-*O-H ↔ (HO)_2_B-*O-*OH + H-O-O-H (23)

However, one additional *in vitro* study [115] was focused on an exploration of the tooth-bleaching efficacies of a 10% (*w/w*) H_2_O_2_ gel, 10% (*w/w*) CP and a 2.0 g quantity of peroxoborate towards human primary maxillary central incisors which were discolored with freshly collected human blood. From the results acquired, it was concluded that all the bleaching agents applied were effective at 7- and 14-day post-treatment time-points (*p* = 0.013), and that the 10% (*w/w*) H_2_O_2_ gel treatment exerted a greater tooth-whitening activity than those of the 10% (*w/w*) CP and peroxoborate formulations applied.

Recently, Tran et al. [116] reported on monitoring of the time-dependence of H_2_O_2_ release from peroxoborate and its depletion thereafter *in vitro* in order to relate findings arising therefrom to those occurring during the internal bleaching of discolored teeth with the sodium salt of this oxidant. The requirement for such a study was that the replacement time intervals for this tooth-whitening agent have usually been based on preferential clinical selections, i.e., those without a sound scientific foundation. From the results acquired, it was concluded that frequent replacements of peroxoborate may not be required in view of the persistence of H_2_O_2_ for durations of at least 28 days. However, it may be that the low levels determined at the later time-points will be insufficient to continue peroxoborate’s favourable tooth-bleaching actions. Critically, these researchers employed a less than reliable method for the quantitation of H_2_O_2_ levels, i.e., a spectrophotometric system involving the oxidation of an Fe(II)-thiocyanate complex to its Fe(III) form, and hence analysis results arising therefrom are not likely to be sufficiently reliable, especially at low or very low H_2_O_2_ analyte levels. Moreover, unfortunately it is entirely conceivable that peroxoborate itself will also respond positively to this spectrophotometric assay system, and therefore, without any evidence that peroxoborate was completely hydrolyzed to borate and H_2_O_2_ in their analysis solutions (Equation (1)), it appears likely that both this target analyte and peroxoborate were being simultaneously monitored in this assay. A further important point is that no allowances were made in this investigation for the consumption of these oxidants by endogenous peroxide scavengers, and therefore the above 28 day replacement time estimate is certainly not extrapolatable to the situation *in vivo*, and may indeed be considerably lower.

In 2006, our research group performed a spectrophotometric determination of the effectiveness of two peroxoborate-containing products to bleach MBPs derived from the reaction of L-lysine with an equimolar concentration of D-glucose. at 60 °C and pH 7.00 [117]. The first of these products was an oral rinse (based on a peroxoborate/glycerol admixture), whereas the second was a reference tooth-whitening formulation with a glycerol: peroxoborate molar ration of 14.9:1. Treatment of these MBPs with increasing volumes of these products demonstrated volume-dependent decreases in intense melanoidin absorption bands located in the visible region of the electromagnetic spectrum, i.e., up to 600 nm, following a sufficient period of equilibration. The time-dependence of these reactions was also monitored, and the rate of these processes was found to increase with increasing volumes of both products added, as expected. These data therefore demonstrated that peroxoborates and their glycerol esters present in the products evaluated successfully bleach MBPs which serve as model compounds for extrinsic tooth discoloration. However, it should be noted that aqueous solutions derived from these product peroxoborate species also contain significant levels of H_2_O_2_ arising from their dissociation (Equation (1)), and therefore the results acquired in this model system are likely to include MBP-bleaching contributions from this liberated oxidant. Nevertheless, a plausible explanation for the tooth-whitening properties of peroxoborates and their ester adducts is their direct oxidative attack on >C=C< units present in the brown-colored Maillard reaction products/melanoidins [83] to form colorless or less intensely-colored epoxide products (Equation (15)). Intriguingly, the kinetic-spectrophotometric assessment strategy applied in the featured study can be effectively utilized to assess the bleaching activities of peroxide-containing oral health and tooth-whitening formulations *in vitro*.

Finally, Chng et al. [118] tested the abilities of 30% (*w/v*) H_2_O_2_, an aqueous solution of sodium peroxoborate, and an admixture of sodium peroxoborate with 30% (*w/v*) H_2_O_2_ tooth-whitening treatments to adversely influence the ultimate tensile strength, micro-punch shear strength, and microhardness of bleached human dentin; results acquired were then compared with those from a negative water control group. A total of n = 41 intact premolars were primarily root canal-treated and each was then then randomly placed in one the above four treatment groups (tooth-whitening agents were sealed within pulp chambers in accordance with their clinical application). Extracted teeth were sectioned, and dentin samples acquired therefrom then underwent biomechanical assessments. From this study, the researchers concluded that although intra-coronal bleaching conducted with 30% (*w/v*) H_2_O_2_ and peroxoborate, either alone or as a composite admixture, gave rise to a weakening of dentin, when used alone the H_2_O_2_ treatment was more damaging than both the peroxoborate approaches tested.

In summary, an overall and extensive survey of the scientific literature has revealed that in some reports peroxoborate-containing products show an improved tooth-whitening activity over those consisting of H_2_O_2_ or CP, although others indicate that the reverse is the case. However, peroxoborate appears to offer a greater tooth-penetrative ability than H_2_O_2_, and when applied alone or in combination with CP, exhibits a lower level of deleterious side-effects, observations consistent with its less harsh therapeutic and cosmetic outlook.

## 8. Mutagenic and Genotoxic Potential of Peroxoborate when Employed at High Concentrations for Tooth-Whitening Purposes?

In 1989, Seiler [75] explored and documented the mutagenic properties of what is described as sodium perborate; however, assuming that dilute aqueous solutions of this agent were employed for this study, then it would appear that the majority of the peroxide was present as free H_2_O_2_, with very limited quantities of [B(OH)_3_(O_2_H)]^−^ available, and perhaps only extremely limited traces of [[(HO)_3_BOOB(OH)_3_]^2−^ and other species present (Section 2). Nevertheless, this researcher found that, as observed with H_2_O_2_ [119], peroxoborate did indeed exert mutagenic properties in a series of *in vitro* testing systems, including one assay which was customized to monitor DNA damage by this oxidant. Curiously, a significant oxidation of thymidine was observed at a temperature as high as 80 °C, whereas only a small level of oxidative conversion of this substrate was observed at 40 °C. Although peroxoborate gave rise to point mutations in the TA100 and TA102 strains of *Salmonella typhimurium*, it did not in the TA98 strain. Incubation of this oxidant with a rat liver mammalian auxiliary metabolic system completely eliminated the mutagenic activity observed, and this would, of course, be expected in view of the availability of a range of direct H_2_O_2_ scavengers (including the enzyme catalase, and low-molecular-mass α-keto acid anions such as pyruvate, oxaloacetate and 2-oxoglutarate) therein, together with many biomolecules with the ability to scavenge any ^●^OH radical derived from peroxoborate or peroxoborate-liberated H_2_O_2_ via pseudo-Fenton or Fenton processes.

Additionally, widespread chromosomal damage in the CHO-K1 strain of Chinese hamster ovary cells was demonstrated on treatment with peroxoborate, and an unusual prevalence of chromosome rearrangements was observed. Hence, this oxidant, (or more likely H_2_O_2_ arising therefrom) was regarded by the author as a direct-acting *in vitro* mutagen.

The clear differences in mutagenic behaviour notable between the above observations and those reported in [62] above can conceivably be rationalized by considerations of: (1) the precise molecular nature of peroxide equivalents available in the above tooth-bleaching gel formulation, i.e., [B(OH)_3_(O_2_H)]^−^, [(HO)_3_BOOB(OH)_3_]^2−^, and presumably higher and much higher contents, respectively, of peroxoborate-glycerol ester species in view of the large excess of glycerol available in the product tested in [62]—such molecular heterogeneity, including any ‘free’ H_2_O_2_ therein is likely to attenuate the bioactivities of this peroxide source; and (2) the ability of the glycerol ester moiety to scavenge any ^●^OH radical inadvertently generated from the catalytic degradation of peroxoborate and H_2_O_2_ by adventitious trace levels of iron and copper ions present in the product itself, or in the cell culture media utilized. Damage to DNA base adducts is predominantly inflicted by the attack of ^●^OH radical on its purine and pyrimidine base adducts, since H_2_O_2_ itself is only poorly reactive towards these substrates.

## 9. Ability of a Peroxoborate Oral Healthcare Product to Attenuate Oral Cavity Volatile Sulfur Compound Levels *In Vivo* and hence Combat Oral Malodor

A very high proportion of the human adult population are afflicted with oral malodor (halitosis, bad breath), which is an aesthetically disturbing and recurring disorder [120]. Cases of this socially distressing condition are attributable to microbial putrefaction at anaerobic loci within the oral cavity [121,122], a process giving rise to the adverse production of highly malodorous VSCs [120,123,124]. There are many reasons why halitosis of an oral etiology may be triggered, and these include excessive bacterial colonization of the tongue, limited salivary flow rates, the perpetuation of periodontal diseases, the use of unclean dentures, and poor or inappropriate dental restorations [120,123].

Since peroxoborate exerts powerful oxidizing activities towards thiol (Equations (9)–(12)) and thioether compounds (Equations (13) and (14)), and also displays useful microbicidal actions [9,10], in principal it should have the capacity to exert valuable therapeutic actions against oral malodor. However, to date, there are little or no reports available on the ability of oral healthcare products containing this oxidant and its molecular heterogenors in aqueous solution, or its polyol ester adducts, to combat halitosis. Therefore, in this section, we provide results from a study performed for the first time by our research team to explore the clinical effectiveness of a peroxoborate-containing oral rinse product [Ardox-X™] against halitosis using a portable gas-chromatographic system, an analytical tool which can determine parts-per-billion (ppb) levels of 3 different VSCs (H_2_S, CH_3_SH and (CH_3_)_2_S) simultaneously in air directly sampled from the oral cavity. These VSC determinations were made before (0.00 h.), and at selected diurnal time-points after treatment of participants with this oral rinse formulation in the recommended manner at 0.33, 1.33, 2.33, 4.00 and 6.00 h. post-treatment. Results acquired were compared with corresponding measurements made after the same participants had rinsed with a H_2_O placebo control in place of the oral rinse in a randomized cross-over experimental design.

This investigation involved 30 non-smoking human volunteers (14 male, 16 female); when recruited, participants were provided with a participant information sheet, and if agreeing to take part in the investigation, were subsequently required to sign a University Research Ethics Committee consent form (participants were excluded from the investigation if they had any serious or chronic medical condition such as diabetes, cardiovascular diseases or cancer, or any other condition which precluded their participation in the trial). Subjects receiving any form of medication during the seven-day period prior to the first testing day were excluded from the investigation. Participants were also instructed not to receive any form of medication during the three sampling test days of the trial. All ethics considerations were fully consistent with those of the Declaration of Helsinki of 1975 (revised in 1983).

Participants were required to rinse with 10 mL volumes of the above commercially-available mouth rinse product for a period of 30 s for a single pre-specified trial day. Each participant also rinsed with an equivalent volume of tap water which served as a negative placebo control on a single corresponding day either prior or subsequent to the oral rinse treatment protocol; the second treatment phase was only performed following an acceptable ‘washout’ period (detailed below). The first (baseline) measurement was made at 09.00 am, and all participants were required to agree to avoid their early morning breakfast meal (and, of course, all further oral activities such as eating, drinking, tooth-brushing, etc.) during the period between awakening in the morning and the first (baseline zero-control) VSC determination on each of the two days in which they were involved in the investigation. Administration of the oral rinse or water placebo treatments to each of the 30 participants was ‘staggered’ throughout time, and the ‘washout’ period between each of the 2 treatment regimens administered (i.e., oral rinse *versus* water control) was three days. During these ‘washout’ periods, all participants resumed their normal oral healthcare activities. Randomization of the cross-over order for the n = 30 participants was achieved using a computerized random number generator.

For each of the above clinical oral cavity VSC concentration datasets, analysis-of-variance (ANOVA)-based experimental designs were employed for this investigation; ANOVA rather than analysis-of-covariance (ANCOVA) models were selected in view of the assumption of linear relationships between dependent variables and quantitative predictor covariables required for the latter model, and clearly visible deviations from this in view of the non-linearities of the responses of all oral cavity VSC levels to post-treatment time-points observed. Hence, for this analysis, the sampling time-point variable was treated as a qualitative rather than an quantitative covariable. The primary, major experimental design (model I) was classified as mixed-model, three-factor system with treatments (one oral rinse and the water placebo control) and time-points at which the measurements were made being fixed effects at two and six levels respectively, and participants (n = 30 in total) representing a random effect, Equation (24)). In this Equation, µ represents the null mean value in the absence of all sources of variation, and M*_i_*, T*_j_*, P*_j_*, MT*_ij_*, MP*_ik_*, TP*_jk_* and e*_ijkl_* represent the ‘between-treatments’ (fixed effect), between-sampling time-points’ (fixed effect), ‘between-participants’ (random effect), treatments × sampling time-points, treatments × participants, and sampling time-points × participants interaction effects, and unexplained error sources of variation, respectively. The three second-order interaction effects were also incorporated into this design in order to explore any differential responses of participants to the treatments applied, and differences between the two treatment regimen responses with regard to the time-point dependencies of VSC concentrations observed post-treatment. The VSC-neutralizing capacity of the mouth rinse product tested was rationalized with special reference to the VSC-oxidizing and bactericidal activities of its active peroxoborate and peroxoborate-glycerol ester constituents.

y*_ijkl_* = µ + M*_i_* + T*_j_* + P*_k_* + MT*_ij_* + MP*_ik_* + TP*_jk_* + e*_ijkl_* (24)

**Figure 5:**
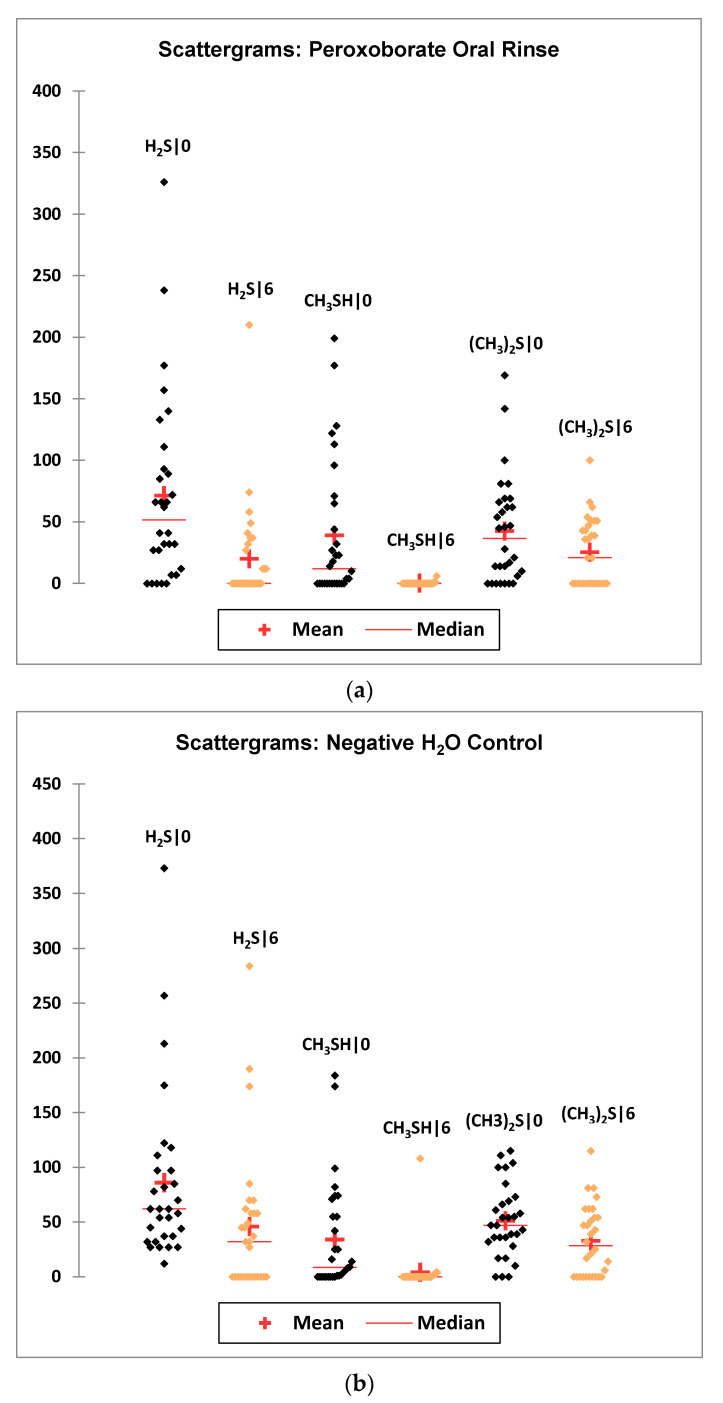
Scattergrams of oral cavity H_2_S, CH_3_SH and (CH_3_)_2_S level datasets at the 0.00 and 6.00 h. time-points for (**a**) the peroxoborate oral rinse treatment regimen, and (**b**) the negative water control groups. Mean and median values for these groups are provided. Abbreviations: H_2_S|0 and H_2_S|6, H_2_S concentrations (ppb) at the 0.00 and 6.00 h. time-points respectively, with the same abbreviations for CH_3_SH (CH_3_SH|0 and CH_3_SH|6) and (CH_3_)_2_S ((CH_3_)_2_S|0 and (CH_3_)_2_S|6).

**Figure 6:**
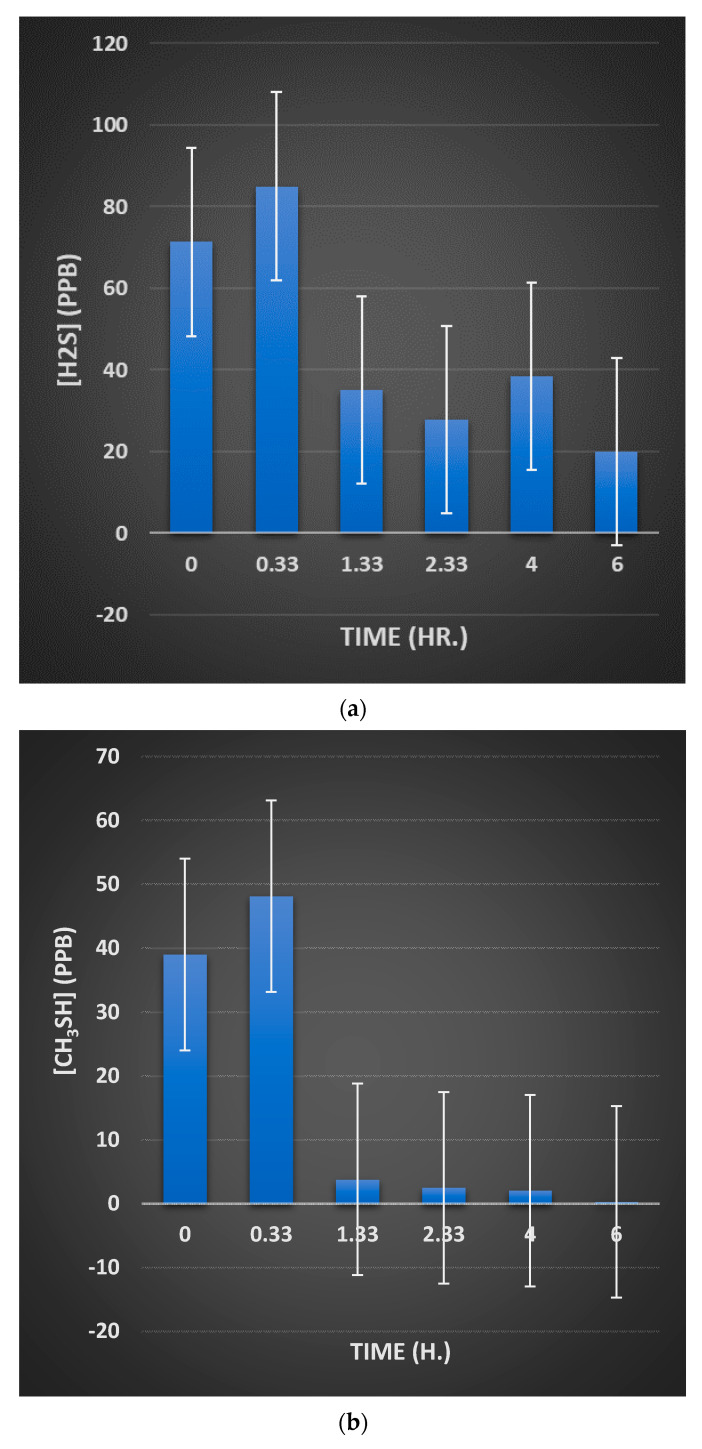

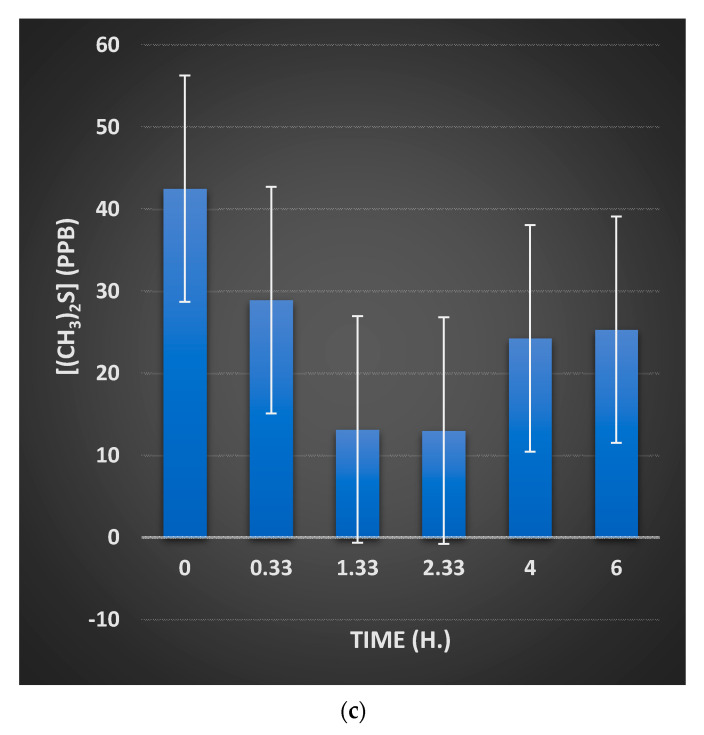
Bar diagram representations of mean±95% confidence intervals (CIs) oral cavity volatile sulfur compound (VSC) concentrations at each study time-point (0.00–6.00 h.) for (a) H_2_S, (**b**) CH_3_SH and (**c**) (CH_3_)_2_S (ANOVA model 3). The 95% CIs include contributions from the ‘between-participants’ and the participant x treatment and participant x sampling time-point sources of variation, and hence they appear much wider than those computed from the error (residual) variance component alone.

Data acquired demonstrated that the peroxoborate oral rinse formulation gave rise to very highly significant, time-dependent reductions in the oral cavity levels of all three VSCs evaluated, and for H_2_S and (CH_3_)_2_S, these effects were substantially greater than those observed with the negative water control (*p* = 3.14 × 10^−3^ and 1.56 × 10^−4^, respectively), albeit less so for CH_3_SH. Moreover, a model I contrast comparison of the magnitudes of the mean VSC concentration differences observed between the 0.00 and 6.00 h. post-treatment time-points revealed that these were significantly greater for the oral rinse treatment group than those for the water control, for both H_2_S and (CH_3_)_2_S (*p* = 2.34 × 10^−4^ and 3.10 × 10^−2^, respectively). Notwithstanding, differences observed between these two treatment groups for CH_3_SH concentrations were much less pronounced, with the negative water control rinse also appearing to exert a significant ‘dampening’ effect on its oral cavity air concentrations. Despite this, at the 1.33 h. time-point, the oral rinse application gave a 90% decrease in this VSC’s level, whereas only a 56% reduction for it was observed with the water control (*p* = 0.027, Bonferroni-corrected ANOVA contrast analysis). These data are shown in Figure 5 and Figure 6. For H_2_S, however, there was also a significant participant x treatment interaction effect (i.e., the VSC level response to treatments differed significantly between-participants), *p* = 1.04 × 10^−2^; indeed, for the peroxoborate rinse treatment, it was clear that a high proportion of participants responded well to it, with major reductions observed in oral cavity levels of this VSC, whereas responses for others were less so, or not significantly affected, by its use.

Notably, for CH_3_SH there was an extremely highly significant treatment x time-point interaction effect (*p* = 9.1 × 10^−7^), and this clearly demonstrated differences in the nature of the time-dependent response of participant VSC levels observed following oral rinse treatment when compared to that seen for the H_2_O control; the major significant difference found between its mean concentrations at the 1.33 h. time-point noted above largely contributes towards the significance of this interaction effect.

As expected, differences between the post-treatment time-points were also very highly significant (*p* < 10^−6^ for H_2_S and CH_3_SH, and 1.17 × 10^−6^ for (CH_3_)_2_S), as was the participant × post-treatment time-point interaction effect for H_2_S and CH_3_SH (*p* = 8.00 × 10^−3^ and 1.07 × 10^−4^, respectively), observations confirming differential diurnal participant responses of these VSCs to the treatments. Notably, there were also significant or highly significant differences observed between study participants (*p* = 2.50 × 10^−6^, < 10^−6^ and < 0.05 for H_2_S, CH_3_SH and (CH_3_)_2_S, respectively), as might indeed be expected.

Multivariate analysis-of-variance (MANOVA) offers greater statistical power when two or more of the dependent variables involved, in this case VSC concentrations, are correlated, and in such cases is valuable for the identification of effects that are of a lower magnitude than those detectable by ANOVA alone. Therefore, this MANOVA strategy was performed here with all 3 VSCs as dependent variables, and results acquired confirmed that there were very highly significant differences ‘between-treatments’ and ‘between-sampling time-points’ (*p* = 2.92 × 10^−4^ and < 10^−6^, respectively, using a combination of Wilks’ (with Rao’s approximation), Hotelling-Lawley’s, Pillai’s and Roy’s tests), but not ‘between-participants’. Furthermore, although the participant x sampling time-point interaction effect was found to be statistically significant (*p* = 0.021), the other two first-order interactions considered were not.

Hence, for H_2_S, the peroxoborate-containing oral rinse formulation exerted a significant or very highly significant VSC-neutralizing activity, which was of a much greater magnitude than that observed with the water control rinse, as expected. Moreover, the Ardox-X™ product was also found to be very effective at diminishing oral cavity concentrations of (CH_3_)_2_S, which arises from a non-oral source [123]. Although this exogenously-mediated retention of mean oral cavity VSC concentrations at lowered values was prolonged for a period of 6.0 h. for H_2_S (and CH_3_SH), the minimal mean level of (CH_3_)_2_S was observed at the 1.33 and 2.33 h. time-points, after which it increased again at the 4-6 h. points, but was still maintained at significantly lower concentrations than those observed at the 0.00 h. starting-point. Potentially, these differences in response observed may be explicable by the differential sources of these two VSCs, i.e., oral environment for H_2_S, and blood for (CH_3_)_2_S.

Mechanisms for the peroxoborate-mediated oxidative consumption of H_2_S and CH_3_SH presumably involve the reaction sequences shown in Equations (9)–(12), whereas that for (CH_3_)_2_S will likely proceed through the routes provided in Equations (13) and (14). However, the abilities of peroxides, including peroxoborates, to kill bacterial sources of these VSCs (gram-negative anaerobes located on the tongue dorsum via a post-nasal drip [125]), and/or oxidatively attack the enzymes therein involved in their adverse generation, are alternative possible mechanisms. Indeed, the VSCs analysed arise from the microbial putrefaction of cysteine- and methionine-containing proteins, and being thiol and thioether species respectively, these amino acids are also susceptible to oxidative attack by peroxoborate and other peroxides. Optimal putrefaction activity occurs in anaerobic oral environments with limited carbohydrate concentrations, and at physiological pH values and temperatures [124].

Of critical importance to the above proposed direct chemical consumption mechanism of peroxoborate action against oral malodor, Deary et al. [31] reported that such species (as an admixture containing a high concentration ratio of borate to H_2_O_2_) reacted almost ten-fold more rapidly than H_2_O_2_ with (CH_3_)_2_S (k_2_ = 3.38 × 10^−1^ M^−1^ s^−1^), whereas the second-order rate constant for its reaction with hydrosulfide anion (HS^−^) was *ca.* three-fold lower than that for H_2_O_2_, which was 2.99 × 10^−1^ M^−1^ s^−1^. This observation may serve to partially explain why the oral rinse formulation tested here was more effective at diminishing oral cavity air (CH_3_)_2_S concentrations than it was those of H_2_S, notably at the 0.33 h. post-treatment time-point. However, such deductions should be made with caution, since an H_2_O_2_ rinse of equivalent peroxide concentration was not also investigated as a positive control in this pilot investigation. Furthermore, H_2_O_2_ is liberated from peroxoborates on their dissolution in aqueous environments (Equation (1)). For the prominent salivary electron-donor SCN^−^, the authors of [31] calculated that peroxoborate reacted with this substrate approximately nine-fold more rapidly than H_2_O_2_ did (k_2_ = 5.0 × 10^−3^ *versus* 5.4 × 10^−4^ M^−1^ s^−1^ respectively). In fact, with the exception of HS^−^ and S_2_O_3_^2−^, all other electron donor substrates explored, including halides such as Br^−^ and I^−^, were found to react an absolute minimum of four-fold faster with H_2_O_2_ in solutions containing excess borate, over those in equivalent media without added borate.

**Figure 7:**
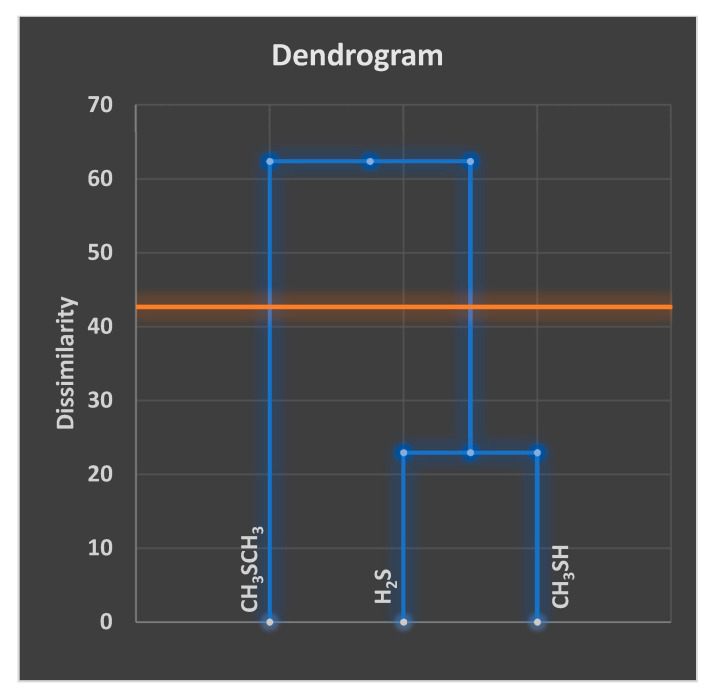
Agglomerative hierarchical clustering (AHC) dendrogram diagram of the 0.00 h. time-point oral cavity VSC level dataset demonstrating a clear distinction of clusters consisting of (1) combined and strongly correlated H_2_S and CH_3_SH concentrations, and (2) (CH_3_)_2_S concentration. The horizontal orange boundary shows the dissimilarity threshold limit for this model.

An agglomerative hierarchical clustering (AHC) analysis was also performed in order to further explore correlations and associations between the VSC concentrations monitored. This analysis was conducted on the zero control dataset only, since the peroxoborate product (and less so water control) treatments evaluated are expected to give rise to a differential consumption level for each of the three malodorous agents monitored, a process which will affect multivariate clusterings. This analysis revealed that the 3 VSC variables were effectively segregated into two clear clusters, the first consisting of highly-correlated H_2_S and CH_3_SH variables, and the second the (CH_3_)_2_S one alone (Figure 7). These distinctive results reflect the sources of these malodorous agents, i.e., both H_2_S and CH_3_SH have an oral environment source, whereas (CH_3_)_2_S arises from extra-oral sources (predominantly blood-borne).

Consistent with these results, Pearson and partial correlation coefficients of VSC levels (albeit for the complete dataset) revealed a very strong correlation between oral cavity H_2_S and CH_3_SH levels (r = 0.58 and 0.57, respectively, *p* < 10^−41^ and < 10^−40,^ respectively), but much lower ones between H_2_S and (CH_3_)_2_S, and CH_3_SH and (CH_3_)_2_S levels.

Therefore, in conclusion, these results provided evidence that the peroxoborate/peroxoborate-glycerol ester oral rinse tested acts as a valuable intra-oral neutralizer and/or oxidative consumer of VSCs, observations which provide a platform for its use in the control of oral malodor. This anti-halitosis efficacy was found to have a longevity of 6 h. or more. These results are also of great clinical significance in view of the known highly toxic actions of VSCs, and their associations with the pathogenesis of periodontal diseases [126]. Intriguingly, it certainly appears that this peroxoborate formulation has the ability to diminish oral cavity concentrations arising from both oral (H_2_S and CH_3_SH) and extra-oral, blood-borne ((CH_3_)_2_S) sources.

## 10. Specificity of the Inhibition of Proteinase Enzymes by Peroxoborates

The possible adventitious inhibition of proteinase enzymes, such as NF-κB, via oxidation of critical methionine residue -S-CH_3_ functions, provides evidence for a favourable peroxoborate-specific and virtual H_2_O_2_-independent mechanism of action for oral healthcare products containing this agent and/or its adducts. Indeed, data are available regarding the reaction of this functional site in subtilisin-type proteinases with peroxoborate rather than H_2_O_2_ itself; this process is much more specific with peroxoborate [127]. This study found that thermitase, subtilisin Carlsberg, alkaline proteinase ZIMET 10,911 and proteinase K were partially inactivated by H_2_O_2_ in the alkaline pH range, but only in the presence of borate or phenyl-boronate. A model to describe this mechanism of inactivation involved an equilibration of both borate and peroxoborate with H_2_O_2_ bound competitively at the enzyme’s active site, and enzyme inactivation arose from the production of a corresponding sulfoxide from the methionine residue located at the active site of these proteinases [128], processes attributable to enzyme-bound peroxoborate species. The same mechanism of inactivation was found for phenyl-boronate present in alkaline H_2_O_2_ solutions.

The above phenomenon is considered to be of much therapeutic importance, especially since such proteinases play a critical role in inflammatory processes, and therefore may provide evidence for the ability of peroxoborate species to exert anti-inflammatory actions in the oral environment.

Furthermore, the possible anti-inflammatory properties of dietary boron [129] should also be considered in the light of the above considerations. In principal, proteinase-inhibiting peroxoborate could be generated from ingested borate *in vivo* via its interaction with phagocytically-generated H_2_O_2_ at sites of inflammation, including those in selected oral environments (Equation (1)). Since this area is beyond the scope of this paper, readers are referred to Ref. [129], and references available therein.

## 11. Consideration of the Roles of H_2_O_2_ and Peroxoborate Species in Triggering or Perturbing Inflammatory Mediators and Vitamins in Periodontal Diseases

In this section, the possible therapeutic actions of oral healthcare products containing peroxoborate species and/or H_2_O_2_ are discussed in the light of the roles and actions of some key periodontal disease mediators, particularly asymmetric dimethyl arginine (ADMA), C-reactive protein and vitamins, the latter including key antioxidants such as hydrophilic ascorbate, and lipophilic α-tocopherol (α-TOH).

Both inflammatory and immunological processes represent key features of periodontal diseases, and these give rise to perturbances in host responses to infection mediated by periodontal bacteria [130]. Indeed, patients with these conditions display upregulated blood serum inflammatory mediators (C-reactive protein, interleukin-6, and prostaglandins, etc.) [131]. Intriguingly, asymmetric dimethylarginine (ADMA), a by-product of continuous protein modification processes within the cytoplasm of all human cells, serves as an endogenous suppressor of the metabolism of nitric oxide (NO^●^), a key free radical species involved in endothelial function and hence cardiovascular health [132]. NO^●^ is involved in endothelium-dependent vasodilation since it mediates the extent of vessel inflammation, vascular tone, and cell proliferation, and attenuates growth factor release [133].

ADMA acts as an inactive substrate for endothelial nitric oxide synthase (eNOS), and may limit NO^●^ generation when present at excessive levels. Indeed, evidence available indicates that ADMA may give rise to the uncoupling of eNOS [134]. Enhanced biofluid ADMA concentrations have been shown in patients with hypertension, hypercholesterolemia, diabetes and renal insufficiency, along with chronic tobacco smokers [135].

Of considerable importance is the notion that, in view of the reactivity of H_2_O_2_ with TMA [44], it is likely that both this oxidant and peroxoborate may directly react with the tertiary-amine function of ADMA to form its corresponding N-oxide, which presumably will be physiologically-inactive. Hence, assuming that this N-oxide oxidation product lacks activity regarding interference with the endothelium-dependent vasodilation via eNOS blockade, such an oxidative inactivation may have important chemopathological consequences in subjects utilising H_2_O_2_ or peroxoborate-containing oral healthcare products. Indeed, Gee and Williamson (1997) [136] monitored the rate of the reaction of H_2_O_2_ of two differing octyl dimethylamines to form their corresponding N-oxides at 23 °C in a mixed isopropanol/water solvent system, and so the oxidation of ADMA *in vivo* by the above oral rinse oxidants is certainly feasible under physiological conditions—experiments to test this are currently in progress in the authors’ laboratories.

However, when present at high oral environment concentrations, as they will be during and immediately following an oral rinsing or tooth-brushing episode with such products, it may be argued that these oxidants will also significantly chemically-modify and hence inhibit eNOS activity. One study has previously demonstrated that eNOS promoter activity is decreased in endothelial cells treated with H_2_O_2_, a process which at least partially involves the inhibition of activator protein 1 (AP-1) activity [137]. This occurs via a suppression of *c-Jun* activity, which in turn gives rise to a reduced level of AP-1 transcription factor binding to the eNOS promoter (*c-Jun* is a protein that is encoded by the *Jun* gene in humans). These researchers also found that the binding of Sp1, a key modulator of basal eNOS transcription, is also responsive to inhibition by H_2_O_2_ [138].

Interestingly, very recently Isola et al. [139] evaluated the impact of gingival health, periodontal diseases, and/or coronary heart diseases (CHDs) on blood serum and salivary concentrations of ADMA. For this purpose, 35, 33 and 35 patients with periodontitis, CHD and both these conditions, respectively, were involved in the protocol, and results acquired were compared to those of 35 healthy control study participants. C-reactive protein (CRP) levels were also determined in these biofluids. From this investigation, researchers involved found that the median levels of both serum and salivary ADMA (the latter normalized to total protein concentration) were significantly greater in the CHD classification and in the combined periodontitis/CHD groups when compared to those with periodontitis alone and healthy controls. In a univariate analysis model, periodontitis, CHD and high-sensitivity CRP (hs-CRP) were found to be associated with serum ADMA concentrations; however, a multivariate (MV) statistical analysis strategy showed that hs-CRP was returned as a highly significant predictor variable (*p* < 10^−3^). This multivariate approach also demonstrated that hs-CRP and educational socioeconomic status successfully predicted salivary ADMA concentrations. Hence, CHD patients, together with those also burdened with periodontitis as a co-morbidity, had significantly elevated ADMA levels over those of healthy control subjects in both types of biofluid explored. These biomarker data provided much evidence for the now well-known interaction of periodontitis with CHDs.

Additionally, ascorbate (vitamin C) and further antioxidants are also critically featured in endothelial function, and may play important roles in the interaction of periodontal diseases with ischemic heart disease (IHD). In view of this important hypothesis, in an elaborate and detailed investigation, Isola et al. [140] also explored linkages between the ascorbate and general vitamin status of blood serum and saliva, and gingival health, periodontitis and/or IHC. In this study, the biofluid sampling approach was very commendable, since the protocol employed involved a 12 h. fasting period for all participants prior to collection; this is an essential requirement for all such investigations, in view of potential interferences arising from dietary ascorbate and other vitamins. Periodontal and clinical indices of groups of 35 or 36 patients with (1) periodontitis alone, (2) IHD alone, (3) periodontitis plus IHD combined, and (4) 36 healthy control subjects were obtained, and concentrations of ascorbate and further antioxidants (α-TOH, β-carotene, lutein and lycopenes) were then determined in saliva and serum samples collected therefrom. Results obtained demonstrated that patients with IHD, and periodontitis combined with IHD, had significantly diminished concentrations of ascorbate in both biofluids monitored when compared to those of healthy participants and patients with periodontitis alone. Moreover, hs-CRP was found to serve as a promising predictor variable for ascorbate levels in both saliva and serum.

In view of its powerful reducing activities, ascorbate is readily oxidized by peroxoborate [33], as indeed it is by H_2_O_2_ [141]. Therefore, it is possible that oral healthcare products containing these peroxide species transiently disturb the ascorbate:dehydro-ascorbate/further ascorbate oxidation product concentration balance within the oral environment *in vivo* according to their use (e.g., oral rinsing episodes with a mouth rinse, or tooth-brushing experience for 30–40 s), frequency of use (daily or otherwise), and the subsequent time period required for the consumption or loss of these oxidants from human saliva or soft oral tissue environments. For the H_2_O_2_ oxidation, both ascorbate and its primary oxidation product, dehydro-ascorbate, generated threonic acid as a product [140]. Data available indicated that the reaction progressed through three major 6-carbon intermediate agents, although one of these, diketogulonic acid, has powerful antioxidant properties.

In view of its powerful lipid peroxyl radical-scavenging activities, α-TOH is a potent antioxidant, albeit a hydrophobic one. In principle, although the oxidation of this phenolic antioxidant with hydroperoxides is a thermodynamically-favourable process, especially if involving the primary generation of peroxyl or hydroperoxyl radical species (ROO^●^ and HOO^●^ respectively), reactions involving hydrophilic H_2_O_2_, and peroxoborates such as that utilized in [35], will be expected to be limited *in vivo* because of the lipophilic nature of α-TOH and its related antioxidants. It should also be noted that α-TOH has the ability to directly regulate mitochondrial H_2_O_2_ generation, and the excessive production of mitochondrial ROS may represent a significant primary event giving rise to tissue damage noted in vitamin E–deficiency syndromes [142]. Hence, the effective regulation of mitochondrial ROS production by this antioxidant appears to attenuate the expression and activation of signal transduction pathways, together with those of further redox-sensitive modifiers, and in this manner exerts a delaying or preventative action regarding degenerative tissue episodes.

The pivotal role of 25-hydroxyvitamin D (VD) in oral health conditions was very recently reviewed by Botelho et al. [143]. Indeed, VD deficiency (VDD) has been linked to a wide range of such disorders, and an impaired biosynthesis of this micronutrient may significantly expedite these. For example, serious dentin and enamel defects arise from an impaired tooth mineralization process, which in turn is determined by VDD; such defects clearly increase risks regarding the induction and progression of dental caries. Furthermore, higher prevalences of periodontitis and gingival inflammation have been linked to VDD [143]. Therefore, the potential deleterious health effects of VDD are critical, manifold, and widespread. Moreover, it appears that VD may be associated with selected oral cancers and osteonecrosis of the jaw [144].

Since Mahmoodani et al. [145] were successfully able to detect and quantitate oxidized vitamin D3 products in a real fortified whole milk powder using a linear trap quadropole (LTQ)-ion trap, Q-exactive orbitrap and triple quadrupole mass-spectrometric method, and successfully determined that the mode of degradation of vitamin D3 was an oxidative deterioration process, it is conceivable that VD may also be susceptible to attack via oxidative assaults mediated by therapeutically-administered H_2_O_2_ or peroxoborate in the oral environment. Indeed, this biomolecule has a total of three olefinic >C=C< units, as does vitamin D3, which may, at least in principle, be oxidized to products containing epoxide functions and/or further products (Section 5.4), processes which may mitigate its biological functions and activities.

Notwithstanding, Jungert and Neuhauser-Berthold [146] recently explored associations between VD and biomarkers of anti-oxidative status in a community-dwelling elderly adult population using a cross-sectional and longitudinal approach; confounding factors, such as body composition, nutrition and lifestyle, were all also considered in this model. These researchers concluded that cross-sectional vitamin D status was inversely related to catalase activity, and that longitudinal vitamin D status served as an effective predictor variable for the total antioxidant status of older adults. Particularly notable is the notion that the maintenance of an adequate vitamin D status may have a beneficial impact on the antioxidant defense system in older adults from a long-term perspective.

## 12. Conclusions

Peroxoborate anion species have chemical and biomolecular reactivities, as oxidants or otherwise, which have some level of distinctiveness from those of H_2_O_2_ alone. As noted above, in general the chemistry and biochemistry of peroxoborate and its chemically-stable ester adducts with polyhydroxy compounds such as glycerol (a common excipient ingredient of many oral healthcare products), and carbohydrates and polysaccharides, have a very broad scope. Indeed, their wide range of chemical/biochemical reactions possible *in vivo* include self-aggregation to form dimers, trimers and possibly higher oligomers; hydrolytic equilibria liberating H_2_O_2_; the generation of novel key reactants such as dioxaborirane; nucleophilic oxidative attack on electrophiles (Equations (3) and (4)), albeit with some preference to act as an electrophile in selected cases; redox reactions; transition metal ion complexation; free radical chemistry; the specific inhibition of proteolytic enzymes such as NF-κB; and their potential abilities to interfere with inflammatory mediator cascades and vitamin/antioxidant functions in periodontal diseases, including likely chemical reactions with ADMA, ascorbate and α-TOH. Some of the above chemical and physicochemical properties may also facilitate peroxoborates’ applications as an alternative, albeit unusual, tooth-whitening product. Moreover, these properties, together with their microbicidal actions, support the employment of peroxoborates for use as general protective oral healthcare agents, and for the combat of oral malodor arising from the adverse generation of VSCs. Of particular merit is the ability of peroxoborates and their ester derivatives to store and conserve oxidizing peroxide equivalents. Therefore, such reactions peculiar to peroxoborates potentially bolsters the case for their employment as novel oral healthcare therapies in clinical practice. However, an intense level of additional investigation is required to further explore this fascinating research area.

Finally, peroxoborate, and indeed its ester adducts, present only a low toxicity and an extended shelf-life; such agents are more stable than preparations containing H_2_O_2_ alone. Indeed, aqueous solutions containing relatively concentrated peroxoborate/peroxoboric acid species, e.g., those employed for use as tooth-whitening or oral rinse products, are much more stable and hence are safer to use than those containing equivalent contents of H_2_O_2_ itself. Notably, when employed as a reagent in synthetic organic chemistry, peroxoborates effectively serve as a valuable alternative for unstable H_2_O_2_ solutions that may pose a significant health and even explosion hazard when present at high concentrations.
